# “Breeding on Mountains” Resulted in the Reorganization of Endophytic Fungi in Asexually Propagated Plants (*Ligusticum chuanxiong* Hort.)

**DOI:** 10.3389/fpls.2021.740456

**Published:** 2021-11-10

**Authors:** Lei Kang, Dongmei He, Hai Wang, Guiqi Han, Hongyang Lv, Wanting Xiao, Zhanling Zhang, Zhuyun Yan, Luqi Huang

**Affiliations:** ^1^State Key Laboratory of Characteristic Chinese Medicine Resources in Southwest China, Chengdu University of Traditional Chinese Medicine, Chengdu, China; ^2^State Key Laboratory Breeding Base of Dao-di Herbs, Center for Post-doctoral Research, National Resource Center for Chinese Materia Medica, China Academy of Chinese Medical Sciences, Beijing, China; ^3^College of Medical Technology, Chengdu University of Traditional Chinese Medicine, Chengdu, China

**Keywords:** *Ligusticum chuanxiong* Hort., asexual reproduction, fungal community, transplanting, reorganization

## Abstract

“Breeding on mountains, cultivation in dam areas” is a unique propagation method for the vegetatively propagated plant *Ligusticum chuanxiong*, including two transplants between the mountain and the dam area. It is well known that the environment can influence the endophytic community structure of plants. However, the change of host endophytic flora caused by transplanting in different places and its influence on asexual reproduction are still poorly understood. We carried out three cycles of cultivation experiments on *L. chuanxiong* and collected stem nodes (LZ), immature rhizomes (PX), medicinal rhizomes (CX), and rhizosphere. High-throughput sequencing was performed to analyze the endophytic fungi in all samples. We observed that the diversity and richness of endophytic fungi in *L. chuanxiong* increased as a result of transplanting cultivation from dam areas to mountains. Local transplantation caused minor changes in the endophytic fungus structure of *L. chuanxiong*, while remote transplantation caused significant changes. Compared with LZ after breeding in the dam area, the LZ after breeding on mountains has more abundant *Gibberella*, *Phoma*, *Pericona*, *Paraphoma*, and *Neocosmospora*. The regular pattern of the relative abundance of endophytic fungi is consistent with that of the fungus in the soil, while there are also some cases that the relative abundance of endophytic fungi is the opposite of that of soil fungi. In addition, there is a significant correlation among certain kinds of endophytic fungi whether in the soil or the plants. We have isolated more gibberellin-producing and auxin-producing fungi in the LZ cultivated in the mountains than that in the LZ cultivated in the dam area. The results of pot experiments showed that the three fungi isolated from LZ cultivated in mountainous areas can promote the development of shoots, stem nodes, and internodes of LZ, and increase the activity of plant peroxidase, catalase, phenylalanine ammonia lyase, and other enzymes. We can conclude that transplantation leads to the recombination of the host endophytic fungus, the more significant the difference in the environment is, the greater the reorganization caused by transplanting. Reorganization is determined by the soil environment, hosts, and the interaction of microorganisms. Remote transplantation is a crucial opportunity to reshuffle the micro-ecological structure of the asexual reproduction of plants, and regulate the growth, development, and resistance of plants, and prevent germplasm degradation caused by asexual reproduction.

## Introduction

In angiosperms, there are two pathways of reproduction: sexual and asexual (vegetative reproduction and apomixis) (Barcaccia and Albertini, 2013; Verhoeven and Preite, 2014; Barrett, 2015). Vegetative reproduction is more commonly represented than apomixis among angiosperm lineages, and reproduction is usually by stolons, rhizomes, tubers, plant buds, etc. It has been estimated that 80% possess some means of reproducing in this manner (Verhoeven and Preite, 2014). Indeed, many terrestrial habitats are dominated by species that reproduce by vegetative reproduction, such as saltmarshes, tundra, grasslands, dunes, and the herbaceous understory of woodlands (Hola et al., 2014). Common vegetative plants in agriculture include potato, ginger, sugar cane, yam, banana, and sweet potato (Thomas et al., 2016; Denham et al., 2020; Tiwari et al., 2021; Wu et al., 2021). Vegetative reproduction also provides several evolutionary benefits, such as avoiding the costs associated with sexual reproduction and a means by which adaptive genotypes can be replicated rapidly after colonization of new environments (Charpentier, 2001; Verhoeven and Preite, 2014; Barrett, 2015). Besides, vegetative reproduction has the potential to interfere with pollination and mating, resulting in reductions in the quantity and quality of offspring, either it is unfavorable for seed set and seed germination, or seedling establishment (Handel, 1985; Barrett, 2015; Van Drunen et al., 2015). The quantity and quality of offspring of vegetatively propagated crops are limited by pathogens (mainly viruses and endogenous pathogens). These pathogens accumulate in successive rounds of vegetative propagation, which severely restricts quantity and germplasm exchange between populations, resulting in catastrophic reductions in amount or extinction, which is called asexual reproduction complication (Perrier et al., 2011; Hola et al., 2014; Ploetz, 2015; Fujimatsu et al., 2018; Hameed et al., 2018; Baebler et al., 2020).

**FIGURE 1:**
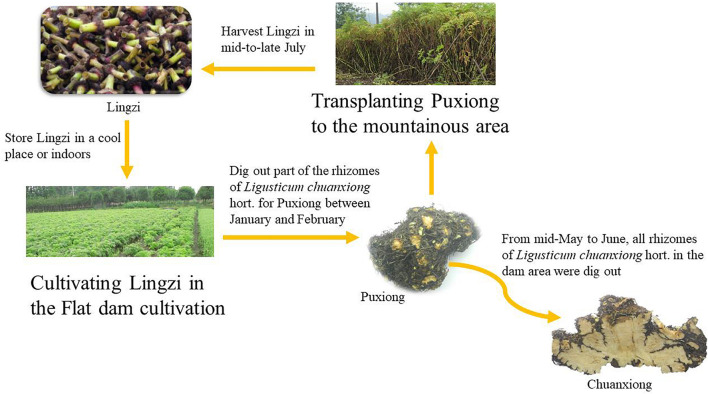
*Ligusticum chuanxiong* cultivation cycle. Lingzi (LZ) refers to the stem nodes of *L. chuanxiong*; PuXiong (PX) refers to the immature rhizome of *L. chuanxiong*; ChuanXiong (CX) refers to the mature rhizome (medicinal materials) of *L. chuanxiong*.

A scientific approach might involve restoring sexual reproduction to avoid the complications of vegetative reproduction (Nyiraneza et al., 2015). An alternative is using an apomictic species (Barcaccia and Albertini, 2013). In addition, effective farming methods such as transplanting are also critical ways to avoid complications of asexual reproduction commonly used in agriculture. It has been shown that potatoes, *Ligusticum sinense*, and *Angelica sinensis* can increase their respective yields by transplanting (Yang and Li, 2006; Zhao et al., 2011; Brazinskiene et al., 2014; Zhang Y. Y. et al., 2016; Iminikuri and Wang, 2018). *Ligusticum chuanxiong* Hort. (LC; Umbelliferae) is an effective medical plant, and has been extensively applied with other Chinese herbal medicines for many years to treat various diseases (Ran et al., 2011); it is widely used in China, Japan, Korea, and other countries, with a history of more than 1,000 years. There are no wild species of *L. chuanxiong*. Cultivated *L. chuanxiong* loses the ability to sexually reproduce and can only reproduce vegetatively. The largest main producing area of *L. chuanxiong* is Sichuan, China. Since the Song Dynasty (800 years ago), the transplanting method of “breeding on mountains and cultivation in dam areas” has been adopted (Figure 1). *L. chuanxiong* that has not been transplanted is susceptible to diseases and insect pests, resulting in reductions in the quantity and quality of offspring (Shan and Hao, 2011). From January to February each year, farmers dig out part of rhizomes in the dam area (400–600 m above sea level, ASL) to remove the stems, leaves, and roots (Puxiong, PX), and transplant them to the mountains (1,000–1,500 m ASL) for breeding (expanded stem node); the remaining rhizomes are left in the dam area for cultivation of medicinal rhizomes. The rhizomes of the plants in the dam area swell and can be used as medicinal rhizomes (Chuanxiong, CX). In May, the stem nodes of the plants in the mountains swell (Lingzi, LZ) and are used as a propagation material in late July, then the stem nodes are transplanted again to the dam area for recycling cultivation (Zhao and Li, 1982). The asexual reproduction process of “Breeding on mountains” is an essential guarantee for the quality and provenance of *L. chuanxiong.* However, the scientific mechanism that transplanting avoids the complications of asexual reproduction still lacks research.

Plants provide a multitude of niches for the growth and proliferation of a diversity of microorganisms. These microorganisms can form complex co-associations with plants and have essential roles in promoting the productivity and health of plants in natural environments (Mansfield et al., 2012; Qiu et al., 2012; Ma et al., 2013; Mendes et al., 2013; Brader et al., 2017; Poveda, 2020; Izquierdo-Garcia et al., 2021; Rasool et al., 2021). Although some members of microorganisms are pathogenic (Mansfield et al., 2012; Ma et al., 2013; Mendes et al., 2013), there are also other microbial communities associated with their hosts that have been shown to promote plant growth (Olanrewaju et al., 2017; Poveda et al., 2021), nutrient uptake (Andrade-Linares et al., 2021), pathogen resistance (Ab Rahman et al., 2018; Duran et al., 2018), and abiotic stress resistance (Qiu et al., 2012; Ma et al., 2013; Izquierdo-Garcia et al., 2021; Trivedi et al., 2021). In agriculture, direct micro-ecological regulation is performed to reduce the complications of plant asexual reproduction. For example, fumigation and manipulation of soil microbial communities can be carried out to control soil-borne diseases of potatoes and increase their quantity (Nyiraneza et al., 2015). Inducing the rhizosphere microbiome by biofertilizer application can be performed to suppress banana *Fusarium* wilt disease (Zhang et al., 2011; Fu et al., 2017; Papik et al., 2020). However, it is not yet known whether transplanting has played an indirect “micro-ecological regulation” effect, which improves the germplasm of plants.

This study aimed to evaluate the impact of transplanting on the endophytic fungi community structure of *L. chuanxiong*. We analyzed differences in the community structure of endophytic fungi during the three cultivation cycles and verified the results of two matches of control breeding experiments and through the result of the fungal isolation experiment of the control experiment and the result of the pot experiment to verify the feedback of endophytic fungi to its host. The scientific mechanism behind transplanting in vegetative reproduction is expected to be explored, which provides a scientific reference for slowing down the complications of other economic plants in asexual reproduction and maintaining the quality of economic plants (Caracciolo and Terenzi, 2021).

## Materials and Methods

### Materials

**FIGURE 2:**
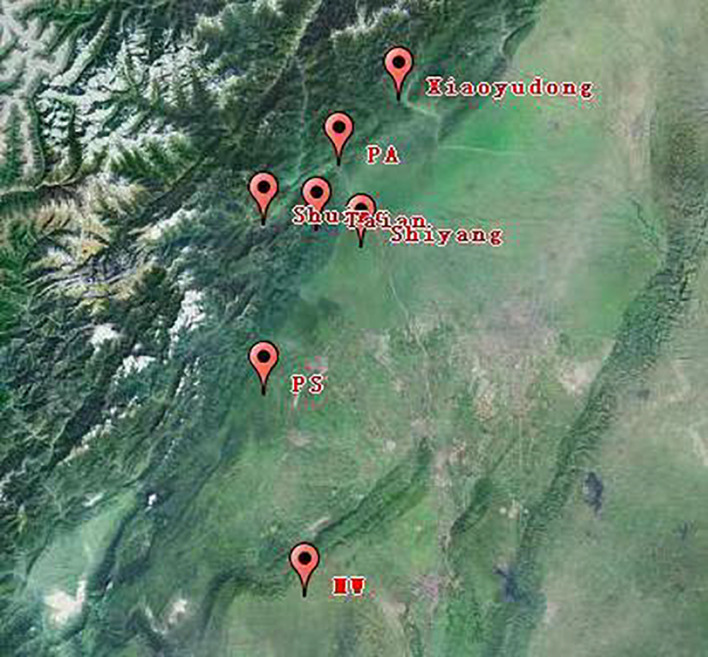
Location map of the study site.

The research consists of three parts. The first part is the “breeding on mountains – cultivation in dam areas” cyclic cultivation experiment. Breeding was carried out in three mountainous areas, namely, Shuimo (1,073 m ASL), Xiaoyudong (998 m ASL), and Taian (773 m ASL) (Figure 2); all LZ were transplanted to the same dam area (Shiyang, 592 m ASL) for the cultivation of medicinal rhizomes. LZ, PX, and CX were collected according to the transplanting sequence and cultivated for two rounds. After ordering all the samples, we collected medicinal rhizomes from four different producing areas (PS, MW, PA, and PD 500–600 m ASL) for verification and analysis (Supplementary Table 1).

The second part is a control experiment of breeding in mountains and breeding in the flat dam. Breeding was carried out at the same time in the mountain of Shuimo (1,073 m ASL) and the dam area of Shiyang (592 m ASL). After harvesting, LZ was transplanted to Shiyang for medicinal rhizome cultivation. According to the breeding place, samples of LZ on the mountain and LZ in the dam area, CX samples, and corresponding soil samples were collected. The soils used in the two parts of the experiment were farming soil of local farmers. During the whole experiment, no fungicide, pesticide, or any form of fertilizer was applied (Supplementary Table 2).

The third part is a pot experiment. We collected LZ from Shuimo and Shiyang to separate fungi and to verify the ability of the separated fungi to secrete gibberellins and auxin. Then, we selected three strains that could produce both IAA and GA at the same time, and the selected three strains were isolated only in the LZ from a mountainous environment. Then, the three strains were cultured in suspension shaking for 7 days. After that, we adjusted the concentration of the spore suspension to 5 × 10^8^ CFU and irrigated the PX that grew in a sterile substrate (humus: perlite, 1:1) with consistent growth. Meanwhile, a blank control group was established to ensure the validity of the results. Four months later, the growth index (plant height, crown width, number of stem nodes, the diameter of stem node, the diameter of the stalk, coefficient of LZ, root weight, and shoot weight) and resistant enzyme content of *L. chuanxiong* were measured. We repeated 10 times for each group (Supplementary Table 3).

Healthy and fresh plants were collected with the cross method. Rhizomes and stem nodes were collected as required, and the surface of the material was washed with running water and rinsed with sterile distilled water after the surface was disinfected (after scraping off the epidermis with a sterile knife, the surface was disinfected by soaking in 2% sodium hypochlorite solution for 15 min and rinsing five times with sterile water to remove sodium hypochlorite), and it was stored in an ultra-low temperature refrigerator at −80°C after being quickly frozen by liquid nitrogen (the LZ material used to separate fungi was stored in a refrigerator at −20°C). The rhizosphere soil was collected by shaking roots and was put into a sterilized 50-ml centrifuge tube after removing impurities and then quickly frozen in liquid nitrogen and kept at −80°C.

### DNA Extraction and Library Construction

Microbial genomic DNA was extracted from the samples using an HP Plant (Soil) Fungal Kit. The extract was detected by 0.8% agarose gel electrophoresis, and DNA concentration and purity were detected by a nucleic acid analyzer (Nanophotometer P330). We selected qualified samples on dry ice and sent them to Shanghai Meiji Company for high-throughput DNA sequencing (DNA quality requirements: concentration ≥ 50 ng/μl, volume > 60 μl, OD260/280 = 1.8–2, no DNA degradation). Fungal ITS-region (the intergenic transcribed spacer region between the 16S rRNA and 23S rRNA genes) was amplified using the forward primer ITS1F (5′-CTTGGTCATTTAGAGGAAGTAA-3′) and reverse primer ITS2R (5′-GCTGCGTTCTTCATCGATGC-3′) which are specific for fungi. The PCR reaction system included 4 μl of 10 × buffer, 2 μl of 2.5 mM dNTPs, 0.8 μl of forward and reverse primers, 0.2 μl of rTaq polymerase, 0.2 μl of bovine serum albumin (BSA), and 10 ng of DNA in a volume of 20 μl. Cycling parameters included initial denaturation at 95°C for 3 min, followed by 35 cycles of denaturation at 95°C for 30 s, annealing at 55°C for 30 s, elongation at 72°C for 45 s, and final elongation for 10 min at 72°C. PCR products were recovered by electrophoresis using 2% agarose gel. Subsequently, the recovered PCR products were purified using an AxyPrep DNA Gel Extraction Kit (Axygen Biosciences, Union City, CA, United States) and eluted with Tris–HCl. The concentration of the purified PCR products was checked by electrophoresis in 2% agarose gel and quantified using QuantiFluor^TM^-ST (Promega, Madison, WI, United States). Illumina paired-end sequencing libraries were prepared using the purified PCR products. After that, the libraries were sequenced using an Illumina MiSeq PE3000 platform. Negative control was established in each practical step to ensure the validity of the test results.

### Fungal Isolation and Cultivation

For fungal isolation, we took 2 g of fresh and healthy LZ for each part of the plant. The procedure was performed for the two plants independently. To isolate fungal epiphytes, tissues were rinsed with water and surface-sterilized in a sequence of 75% ethanol for 40 s followed by 2% NaClO_2_ for 15 min, and finally rinsed twice in sterile distilled water. Disinfected plant tissues were cut and macerated. The extract obtained was recovered and serial dilutions of 10^–1^, 10^–2^, and 10^–3^ were performed, and 100 μl of each dilution was plated on potato dextrose agar (PDA) (The composition of the PDA medium is as follows: potato infusion 20%, dextrose 2%, agar 2%). All plates were incubated at 28°C for 1 month. The plates were checked daily, and each emerging fungal colony was transferred onto a new PDA plate until axenic cultures were obtained.

### Qualitative Determination of Gibberellins

Pure fungal isolates were incubated in 5 ml of PDB medium, for 10 days at 30°C under dark conditions and agitation (150 rpm); 0.5 ml of culture media was recovered and combined with 0.5 ml of 96% ethanol and 5 ml of a cold mixture of sulfuric acid/ethanol (1:1 v/v). This mixture was incubated at 48°C for 30 min and then exposed to a UV lamp. Samples showing green fluorescence were considered positive for gibberellin production (Salazar-Cerezo et al., 2018).

### Qualitative Determination of and Auxins

Pure fungal isolates were incubated in 5 ml of PDB medium (with L-tryptophan), for 10 days at 30°C under dark conditions and agitation (150 rpm); 0.2 ml of the suspension was placed into a test tube, and an equal volume of Salkowski colorimetric solution (35% HClO_4_:0.5 mol/LFeCl_3_, 35:1 v/v) was added. This test tube was placed at room temperature and the light was avoided for 30 min. Samples showing pink fluorescence were considered positive for auxin (IAA) production (Zhang D. Y. et al., 2016).

### Determination of Soil Physical and Chemical Properties and Resistant Enzymes

Soil physicochemical factor parameters, such as pH, total nitrogen (TN), available phosphorus (AP), available potassium (AK), organic matter (OM), were analyzed according to standard soil-testing procedures (Richardson and Simpson, 2011). According to the methods recorded in the literature (Chen and Wang, 2006), the activities of resistant enzymes, such as superoxide dismutase (SOD), peroxidase (POD), catalase (CAT), phenylalanine ammonia lyase (PAL), and malondialdehyde (MDA), were determined, respectively.

### Data Analysis

Clean reads were obtained by filtering the raw sequences using a microbial ecological quantitative analysis pipeline (QIIME, version 1.9.1, USAU). Low-quality sequences (such as uncertain nucleotide sequences, three nucleotides with a *Q*-value of less than 20, and unmatched barcode sequences) were removed. To obtain valid data, QIIME v1.9.0 is used for quality control, and the Uchime algorithm and gold database are used to remove delusion. These sequences were grouped into operational taxonomic units (OTUs) based on 97% sequence identity using UPARSE (V7.0.1090). Each row was annotated by comparing the Ribosomal Database Project (RDP) classifier (V2.11) against the SILVA (SSU123) database2 using a comparison threshold of 70%. Resampling was carried out with the smallest amount of data in the sample as the standard to make the uniform treatment for each sample. Mothur (version 1.30.2)^1^ is used for diversity analysis. Use R 3.6.0 was used to perform various data conversions. The ggplot2 package is used for population analysis, the stats package is used for Kruskal–Wallis rank sum test and cluster analysis, and the Vegan package is used to calculate Bray–Curtis distance and for PCA, RDA, and CCA analysis. FUNGuild (Fungi Functional Guild (FUNGuild) is used for function prediction. All the figures are made with ggplot2. Significant differences among groups were analyzed using one-way ANOVA, followed by Dunnett’s *post-hoc* test; *p* < 0.05 (marked as ^∗^) was defined as significant, *p* < 0.01 (marked as ^∗∗^) was defined as very significant, and *p* < 0.001 (marked as ^∗∗∗^ or more than ^∗∗∗^) was defined as extremely significant.

## Results

### Cycling Cultivation Experiment on *Ligusticum chuanxiong*

#### Diversity of Endophytic Fungi in Different Growth Stages

**FIGURE 3:**
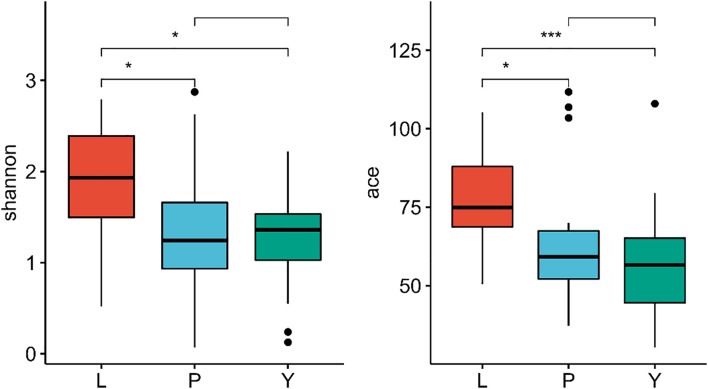
The difference in diversity index of endophytic fungi community in *L. chuanxiong* in different periods. L refers to Lingzi; P refers to Puxiong; Y refers to Chuanxiong. * is defined as significant, *** is defined as extremely significant.

All reads were over 200 bp, and the average length was about 252 bp, which is in line with expectations. The sequence coverage index Good’s Coverage extracted from each sample was more significant than 99.9% (Supplementary Table 4). Microbial diversity was analyzed using the Shannon diversity index, and richness was analyzed using ace indices. The Shannon and ace indices were highest in the LZ (Figure 3), suggesting that the diversity and richness of endophytic fungi are greatest for LZ. There were no significant differences in the Shannon, ACE, or Chao indices between the PX and CX.

**FIGURE 4:**
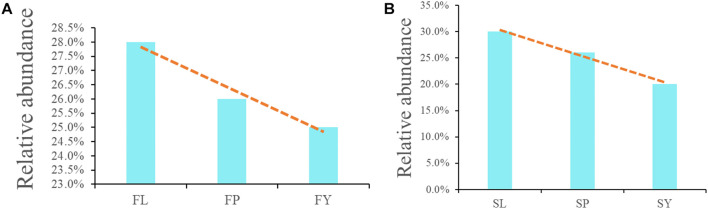
**(A)** Common endophytic fungi of *L. chuanxiong* in the first cultivation cycle; **(B)** Common endophytic fungi of *L. chuanxiong* in the second cultivation cycle. FL refers to the LZ in the first cultivation cycle; FP refers to the PX in the first cultivation cycle; FY refers to the CX in the first cultivation cycle; SL refers to the LZ in the second cultivation cycle; SP refers to the PX in the second cultivation cycle; SY refers to the CX in the second cultivation cycle.

To analyze the differences in the composition of the endophytic fungi of *L. chuanxiong* at different stages, we analyzed the shared fungi of *L. chuanxiong* in different breeding bases and different growth stages. The shared endophytic fungi of PX from different breeding areas accounted for 26% in the first cultivation cycle, which accounted for 26% in the second cultivation cycle, too. As for LZ, in the two cultivation periods, the shared endophytic fungi accounted for 30 and 28.1%. As for CX, however, the shared endophytic fungi accounted for 24.5 and 20.3% (Figures 4A,B).

It shows that the community structure of endophytic fungi in *L. chuanxiong* at different stages is diverse during the two rounds of the cultivation cycle. The shared endophytic fungi of *L. chuanxiong* gradually increased as the PX was transplanted to mountains for the cultivation cycle of LZ and progressively reduced as the PX was transplanted to the dam area to cultivate CX. However, about 20–30% of the fungal OTUs remained stable for a long time in *L. chuanxiong.*

#### Comparison of the Community Structure of Endophytic Fungi at Different Growth Stages

**FIGURE 5:**
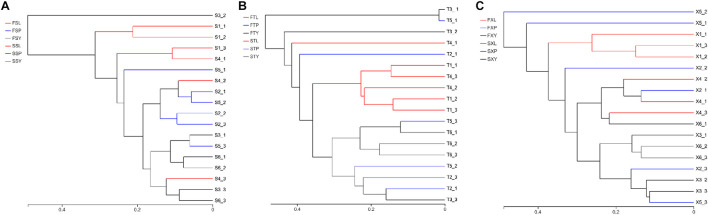
Hierarchical clustering diagram of endophytic fungi in *L. chuanxiong*. **(A)** Refers to breeding on mountains experiment in Shuimo; **(B)** refers to breeding on mountains experiment in Taian; **(C)** refers to breeding on mountains experiment in Xiaoyudong.

The results of the hierarchical cluster analysis show that the LZs, PXs, and CXs from Shuimo can be distinguished and that the samples at each stage tend to gather separately (Figure 5A); the LZs and CXs from Xiaoyudong and Taian tend to aggregate separately, while the PXs from these two origins cannot be distinguished (Figures 5B,C). Generally speaking, the endophytic fungi of LZs and CXs are quite different in community structure, while the endophytic fungi of PXs are similar to both. The results of the PCA (Supplementary Figure 1) show that in the two cultivation cycles, the LZs of the three breeding areas were separated from each other, which tended to be concentrated in the same place, while the PXs and CXs from different breeding areas could not be distinguished. It shows that the cultivation mode of “breeding on mountains and cultivation in dam areas” led to changes in the community structure of the endophytic fungi of *L. chuanxiong*. The most significant change of reorganization occurred in transplanting from the dam to the mountain (and transplanting from the mountain to the dam). The difference in the breeding environment is the most critical factor that causes the community structural reorganization of the endophytic fungi in *L. chuanxiong.*

#### Differential Analysis of Functional Annotations of Endophytic Fungi at Different Stages

**FIGURE 6:**
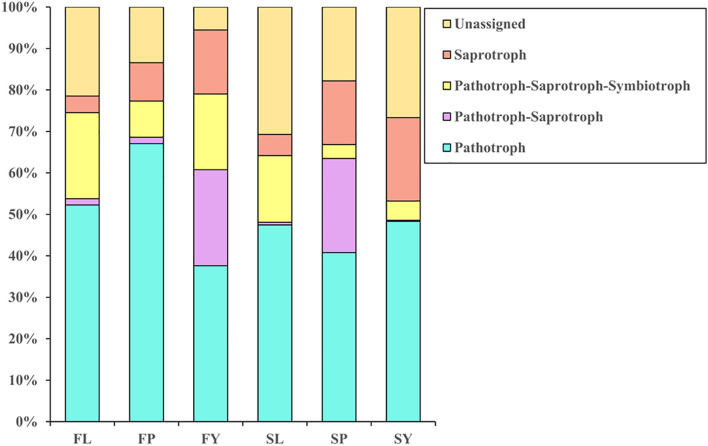
Annotation on the community function of endophytic fungi in *L. chuanxiong.* FL refers to the LZ in the first cultivation cycle; FP refers to the PX in the first cultivation cycle; FY refers to the CX in the first cultivation cycle; SL refers to the LZ in the second cultivation cycle; SP refers to the PX in the second cultivation cycle; SY refers to the CX in the second cultivation cycle.

We have annotated the functions of the endophytic fungi of *L. chuanxiong* (Figure 6). Abundant pathotroph is observed in LZs, PXs, and CXs, and relative abundance is always maintained at a relatively stable size (44∼50%). The ratios of pathotroph-saprotroph-symbiotroph in FL, FP, FY, SL, SP, and SY are 20.6, 8.6, 18.2, 14.8, 3.3, and 4.6%, respectively. In the process of transplanting PX-LZ or PX-CX, the relative abundance of pathotroph-saprotroph-symbiotroph increased, which is the primary functional type of fungi in LZs. The ratios of saprotroph in at each stage were 4.0, 9.1, 15.4, 4.75, 15.1, and 20.0%, which means that during the process of transplanting of FP-FL-SP-SL, the relative abundance decreased first, then increased, and finally decreased again, and gradually increased during the process of FP-FY or SP-SY, which is the primary functional type of fungi in CXs. Pathotroph-saprotroph has a high abundance in the samples from the dam area.

#### Analysis of Differences in Species of Endophytic Fungi at Different Growth Stages

**FIGURE 7:**
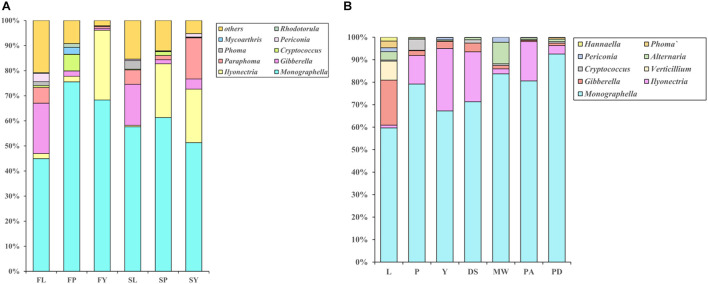
**(A)** Bar diagram of community composition of *L. chuanxiong* endophytic fungi at different growth stages. FL refers to the LZ in the first cultivation cycle; FP refers to the PX in the first cultivation cycle; FY refers to the CX in the first cultivation cycle; SL refers to the LZ in the second cultivation cycle; SP refers to the PX in the second cultivation cycle; SY refers to the CX in the second cultivation cycle. **(B)** Relative abundance of endophytic fungi in *L. chuanxiong*. DS refers to Shiyang town, Dujiangyan, Sichuan province; MW refers to Wansheng town, Meishan, Sichuan province; PA refers to Aoping town, Pengzhou, Sichuan province; PX refers to Xiaoyudong town, Pengzhou, Sichuan province.

A few genera are always the dominant genera at all stages of the cultivation cycle of *L. chuanxiong*, such as *Monographella* (36%). However, the relative abundance of these genera at each growth stage fluctuates regularly (Figure 7A). It shows that the relative abundance of *Pericona*, *Gibberella*, and *Phoma* increases first, then decreases, and finally increases during the process of transplanting of FP-FL-SP-SL. In contrast, the opposite is true for *Cryptococus*. *Gibberella*, *Phoma*, *Pericona* are the dominant genera in LZs (Supplementary Figures 2B–D), and the ratio is 9.4%. During the process of FP-FY and SP-SY, the relative abundances of *Rhodotorala* and *Cryptococus* both decreased, and that of *IIyonectria* increased. *Rhodotorula* and *Cryptococcus* have a high relative abundance in the PXs (Supplementary Figures 2E,F), and the ratios in PX are 1.1 and 4.5%, respectively. *Ilyonectria* is the dominant genus in CXs (Supplementary Figure 2A), and its relative proportions are 18.3%.

#### Analysis of Differences in Species of Endophytic Fungi in Different Medicinal Materials

To learn more about the community structure of the endophytic fungi of *L. chuanxiong*, we analyzed the endophytic fungi in CXs from four other producing areas in Sichuan province. The results are shown in Figure 7B. The genera with higher relative abundance in the rhizomes of various producing areas are also those with higher relative abundance in all the stages of growth in the early cultivation experiment, such as *Monographella*. After transplanting from the PX to CX, the relative abundance of some of the genera increased (such as *Ilyonectria*) or decreased (such as *Gibberella*, *Verticillium*, and *Thanatephorus*, etc.) in CX. In previous experiments, we also found a significant increase or and decrease, respectively, in CX in four other different origins.

The analysis results of endophytic fungi in CXs from four different producing areas in Sichuan province verified the reorganization of fungi, which indicates that the restructuring of endophytic fungi during transplanting will not disappear, regardless of the change in the production area.

### Comparative Breeding Experiment Between Mountains and Flat Dam

#### Diversity of the Fungal Community

To further explore the reasons for the formation of the *L. chuanxiong* micro-ecological structure, we conducted two breeding experiments. In the breeding stage, we dug out PX and transplanted it to the dam area where the CX was cultivated for breeding, which is called “breeding in dam areas.” Simultaneously, we carried out traditional breeding on mountains and completed the follow-up cycle of medicinal material cultivation.

**FIGURE 8:**
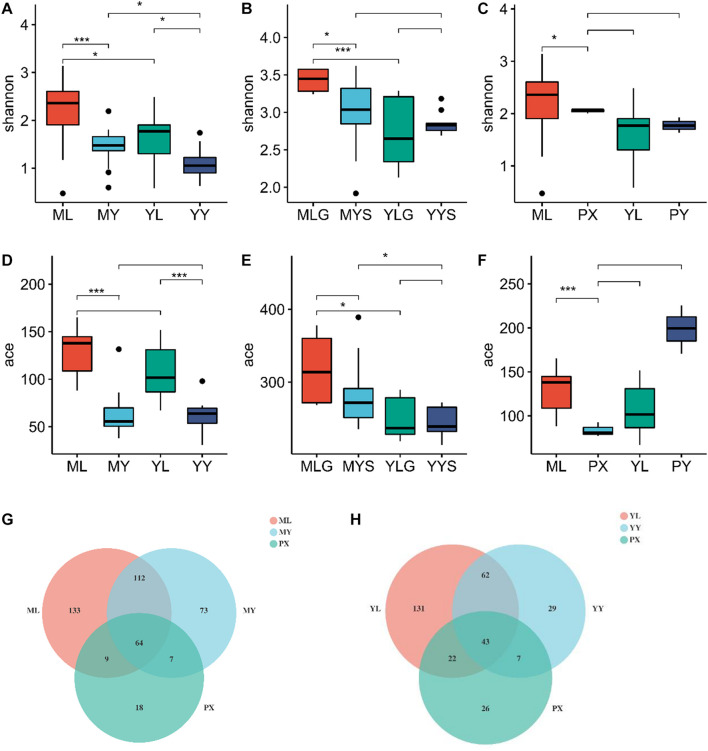
**(A–C)** Shannon index diagram in the comparative breeding experiment; **(D–F)** ace index diagram of comparative breeding experiment between mountains and flat dam. **(G)** The venn diagram of endophytes under M-Y cultivation mode; **(H)** The venn diagram of rhizosphere fungi under Y-Y cultivation mode. ML refers to Lingzi under M-Y cultivation modes; YL refers to Lingzi under Y-Y cultivation modes; MY refers to Chuanxiong under M-Y cultivation modes; YY refers to Chuanxiong under Y-Y cultivation modes; MLS refers to the soil of Lingzi under M-Y cultivation modes; YLS refers to the soil of Lingzi under Y-Y cultivation modes; MYS refers to the soil of Chuanxiong under M-Y cultivation modes; YYS refers to the soil of Chuanxiong under Y-Y cultivation modes. PX refers to the Provenance of Lingzi Puxiong; PY refers to CX without transplantation. PX refers to the Provenance of Lingzi Puxiong; PY refers to CX without transplantation. * is defined as significant, *** is defined as extremely significant.

All reads are over 200 bp, and the average length is about 252 bp, which is in line with expectations. The sequence coverage index Good’s Coverage extracted from each sample is greater than 99.9%. Microbial diversity was analyzed using the Shannon diversity index, and richness was analyzed using ace indices (Supplementary Table 5). The Shannon and ACE indices were higher in the plant than in the soil. The diversity of endophytic fungi in plants showed the same results as the breeding on mountains cycle cultivation experiment, the Shannon and ace indices were highest in the LZs (Figure 3), and there were no significant differences in the Shannon, ACE, or Chao indices between the PXs and CXs. The diversity index of LZs and CXs under the M-Y cultivation mode (breeding on mountains, cultivation in dam areas) is greater than that of the samples under the Y-Y cultivation mode (breeding in dam areas, cultivation in dam areas) (Figure 8). Similarly, for soils, the diversity index of soils on the mountain is greater than that of the soils in the dam area, and the ace index of various soils in the M-Y cultivation mode is greater than that in the Y-Y cultivation mode.

The Venn diagram shows that the shared endophytic fungi of *L. chuanxiong* at each stage under the M-Y cultivation mode accounted for 20.1%. The Y-Y cultivation mode accounted for 16.7% (Figure 8).

#### Differences in Community Structure of Fungi

**FIGURE 9:**
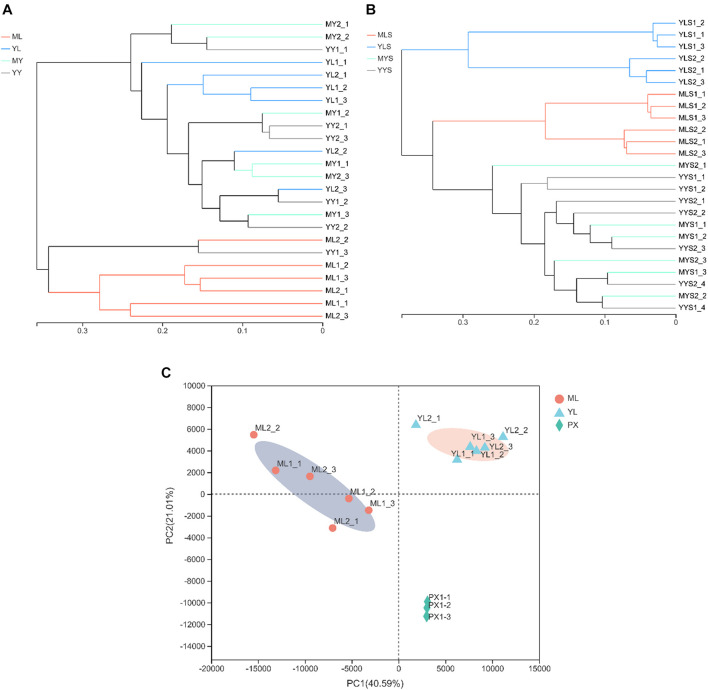
Hierarchical clustering diagram of cultivating Lingzi in the mountains. **(A)** Refers to the hierarchical clustering diagram of plant samples under two cultivation modes; **(B)** refers to the hierarchical clustering diagram of soil samples under two cultivation modes; **(C)** refers to the PCA diagram.

The hierarchical cluster analysis results show that the LZs cultivated in the mountains are different from the LZs and CXs cultivated in the dam area and that the samples cultivated in the dam area cannot be distinguished from either LZs or CXs (Figure 9A), which means that most of the samples grown in mountain areas tend to be different from the samples cultivated in the dam area, and the samples in the dam area are closely related and cannot be distinguished. It shows that the soils from mountains and dam areas are not clustered together and that there is a clear difference, although both of them are soils after sowing. Similarly, it also shows that soils after sowing in the dam area and the soils after cultivating CX are not clustered together and that there is a clear difference, although both of them are located in the same dam area (Figure 9B).

The PCA results indicated that PX and LZ from the two breeding areas were separated. The LZs produced in the same place tended to be concentrated, while the LZs from different breeding areas were separated from each other (Figure 9C). It shows that as long as the transplantation is carried out from the stage of PX to LZ, the community structure of endophytic fungi can be changed.

**FIGURE 10:**
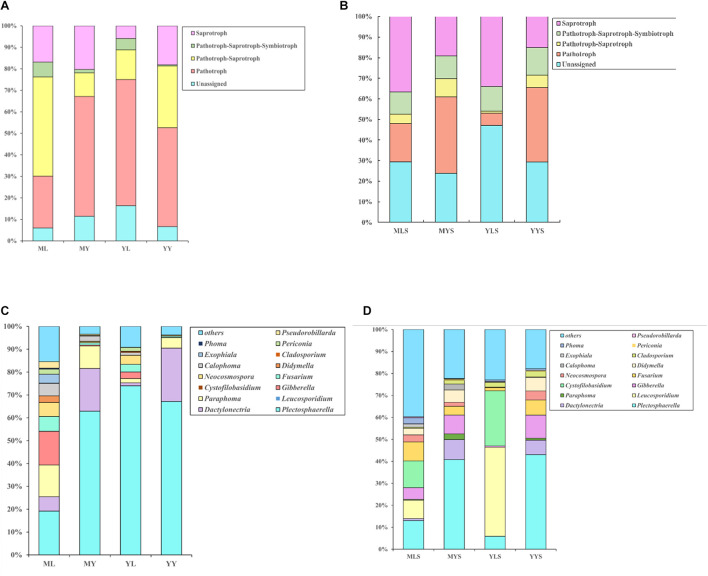
**(A)** Annotation on the community function of endophytic fungi. **(B)** Annotation on the community function of the rhizosphere. **(C)** Bar diagram of endophytic fungi community of *L. chuanxiong* for controlling. **(D)** Bar diagram of rhizosphere community of *L. chuanxiong* for controlling. ML refers to Lingzi under M-Y cultivation modes; YL refers to Lingzi under Y-Y cultivation modes; MY refers to Chuanxiong under M-Y cultivation modes; YY refers to Chuanxiong under Y-Y cultivation modes; MLS refers to the soil of Lingzi under M-Y cultivation modes; YLS refers to the soil of Lingzi under Y-Y cultivation modes; MYS refers to the soil of Chuanxiong under M-Y cultivation modes; YYS refers to the soil of Chuanxiong under Y-Y cultivation modes.

We find the relative abundance of pathotroph-saprotroph-symbiotroph in LZs to be higher than that in CXs, while saprotroph is the opposite (same as the results of the previous experiment). There is more pathotroph in the samples from the mountain than the samples from the dam area, and pathotroph-saprotroph is just the opposite (Figure 10A).

For soils, there are more pathotroph and pathotroph-saprotroph in the soil of CX (MYS, YYS) than in the soil of LZ (MLS, YLS). Saprotroph is just the opposite, while pathotroph-saprotroph-symbiotroph has no difference in each sample of soil (Figure 10B).

#### Analysis of the Differences in Species of Fungi

We analyzed the difference in endophytic fungi in the samples of plants and soils under two transplanting modes (Figures 10C,D). The results showed that LZs (especially the LZs cultivated in the mountains) are dominated by *Gibberella*, *Fusarium*, *Alternaria*, *Calophoma*, *Periconia*, *Didymella*, *Neocosmospora*, *Phoma*, etc., and that the relative abundance in the samples from the M-Y cultivation mode is more significant than that in the samples from the Y-Y cultivation mode. The relationship is ML > YL > MY > YY. Where *Gibberella*, *Didymella*, *Periconia*, and *Alternaria* have a lower relative abundance in LZ’s soils of LZ and have a higher relative abundance in CX’s soils of CX (MYS > YYS > MLS > YLS) (Supplementary Figures 3B,D,F–H), while *Fusarium* has a higher relative abundance in LZ’s farming soils of LZ, which is consistent with the law of the endophytic fungus (Supplementary Figure 3C). CXs are dominated by *Dactylonectria* and *Plectosphaerella*, and the relative abundance of the samples from the Y-Y cultivation mode is greater than that of the samples from the M-Y cultivation mode. The relationship is MY > YY > YL > ML. Similarly, the relative abundance in the soil of CX is also higher (MYS > YYS > MLS > YLS). In addition, regardless of the abundance of the soil, the relative abundance of *Paraphoma* and *Calophoma* in the samples of the M-Y cultivation model was significantly higher than that of the Y-Y cultivation model.

We analyzed the impact of the transplantation of PX on the endophytic fungi of *L. chuanxiong* by one-way ANOVA (Supplementary Figure 4). The results showed that regardless of whether *L. chuanxiong* was transplanted to mountains or adjacent dam areas, as long as the transplanting measures were carried out at the stage of PX to LZ, some endophytic fungi, such as *Gibberella*, *Fusarium*, *Calophoma*, *Periconia*, and *Cercospora*, would continually increase. However, some endophytic fungi, such as *Paraphoma* and *Neocosmospora*, only increased when transplanting from dam areas to mountains, while transplanting on a flat dam did not change significantly. In addition, some genera that are the dominant flora in CX, such as *Dactylonectria*, also increased significantly only when transplanted in the mountains.

#### Association Analysis of Fungi Species

**FIGURE 11:**
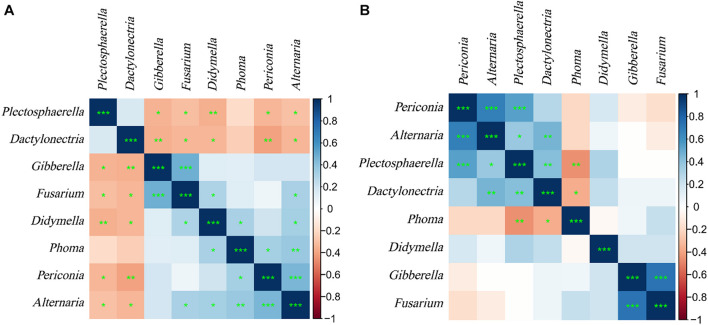
Heat map of correlation coefficient of *L. chuanxiong* endophytic fungi. **(A)** Plant samples. **(B)** Soil samples. * is defined as significant, ** is defined as very significant, *** is defined as extremely significant.

Correlation analysis of common endophytic fungi in the two experiments (Figure 11) showed that *Plectosphaerella* and *Dactylonectria* were significantly negatively correlated with *Gibberella*, *Fusarium*, *Didymella*, *Alternaria*, and *Periconia* in the samples of plants. At the same time, they are significantly positively correlated with *Alternaria* and *Periconia* and significantly negatively correlated with *Phoma* in the samples of soils. *Alternaria* has a significant positive correlation with *Phoma*, *Gibberella*, *Fusarium*, and *Didymella* in plants, but there is no significant difference in soils. Regardless of the samples of plants or soils, *Alternaria*, *Periconia*, *Gibberella*, and *Fusarium* all have significant positive correlations.

#### Environmental Impact on Fungal Communities

**FIGURE 12:**
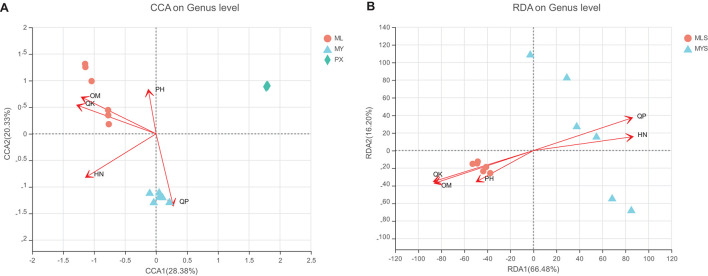
**(A)** Redundancy analysis (RDA) of endophytic fungi. **(B)** RDA of the rhizosphere.

The soil environment has an important influence on plant endophytic fungi. As an important content for us to observe the change mechanism of the endophytic fungus in *L. chuanxiong*, we measured the pH, organic matter, hydrolyzed nitrogen, available phosphorus, and available potassium of the rhizosphere soil of LZ, PX, and CX cultivated in the M-Y model (Table 1). The results showed that whether PX was transplanted to the mountain to cultivate LZ or PX remained in the dam area to cultivate CX, the relative contents of organic matter, hydrolyzed nitrogen, available phosphorus, and available potassium in the rhizosphere increased, and that all of them had significant differences in the three groups of rhizospheres, but that there was no significant difference in pH. The results of RDA and CCA analysis showed that, except for pH, the other four environmental factors had significant effects on the community structure of the endophytic fungi and rhizosphere community of *L. chuanxiong* (Figure 12). Available potassium is the main explanatory factor for the community structure of endophytic fungi in rhizosphere soil, followed by organic matter and available phosphorus. Hydrolyzed nitrogen is the main explanatory factor for the community structure of the endophytic fungi of *L. chuanxiong*, followed by available potassium and organic matter (Supplementary Table 6). It shows that the physical and chemical properties of soil have varying degrees of influence on the community structure of rhizosphere and endophytic fungi.

**TABLE 1:** Soil physical and chemical factors in the rhizosphere of *Ligusticum chuanxiong*.

Group	pH	Organicmatter (%)	Hydrolyzed nitrogen (mg/kg)	Available phosphorus (mg/kg)	Available potassium (mg/kg)
MLG	6.65 ± 0.89	52.06 ± 0.49 a	44.58 ± 0.86 a	172.66 ± 0.13 a	538.7 ± 0.66 a
YPG	6.38 ± 0.89	28.18 ± 0.43 b	24.79 ± 0.40 b	181.05 ± 0.57 b	92.86 ± 0.68 b
YYG	5.65 ± 0.78	32.46 ± 0.50 c	47.42 ± 0.54 c	213.74 ± 0.89 c	228.21 ± 0.34 c

*Means within a column followed by different letters are significantly different (Duncan’s test: P < 0.05).*

The correlation heat map diagram shows that some fungi show the same correlation with environmental factors in the rhizosphere or endophytes (Supplementary Figures 5A,B). For example, *Phoma* has a very significant positive correlation with organic matter and available potassium in rhizosphere and endophytes, while *Rhizoctonia* has a very significant positive correlation with hydrolyzed nitrogen and available phosphorus in rhizosphere and endophytes. There are also some fungi show different correlations with environmental factors between rhizosphere and endophytes. For example, *Gibberella* has an extremely significant positive correlation with organic matter and available potassium, and a negative correlation with available phosphorus in endophytes, while in the rhizosphere, it has a very significant negative correlation with organic matter and available potassium, and a very significant positive correlation with available phosphorus, and so are *Didymella* and *Calphoma*.

### Potted Verification Experiment on *Ligusticum chuanxiong*

#### The Capability of Some Fungal Isolates to Produce Gibberellins and Auxins

As part of our ongoing efforts toward verifying the difference between “Breeding on mountains” and “flat dam breeding,” we have carried out the separation and purification of fungi on the LZ in the mountains and the dam areas and investigated the ability of these fungal isolates to produce secondary metabolites. It is also important to identify these potentially bioactive fungi for a better understanding of the characteristics of the relevant fungal community associated with *L. chuanxiong*. In the search for fungal isolates from *L. chuanxiong* that may have a positive impact on plant development, we performed a general screening to identify gibberellin-producing and auxin-producing isolates using a qualitative method described previously. We tested all the isolates and found that 21 of them were positive for gibberellin production and that 21 of them were positive for auxin by the qualitative test (Supplementary Table 7). Among them, there were 14 GA-producing strains and 13 IAA-producing strains isolated from LZ produced in the mountainous areas, Seven GA-producing strains and 9 IAA-producing strains were isolated from LZ in the dam area.

#### Determination of Growth Indicators

To investigate the impact of LZ core flora on the growth of *L. chuanxiong*, we focused on the three strains that can produce IAA and GA at the same time and are isolated only in the mountain environment. They were identified as *Fusarium moniliforme* (FM), *Fusarium avenaceum* (FAV), and *Fusarium proliferatum* (FP) (He, 2016). All three strains are classified as *Fusarium*, but on the sexual stage they are classified as *Gibberella*, and they are the core flora of *L. chuanxiong*. The production of gibberellins was quantitatively determined by a method described previously. The suspensions of the three fungi were irrigated and inoculated into the potted plants of *L. chuanxiong*. After 4 months of growth, the growth indicators were measured. The results showed that, compared with the CK group (blank control group), the FM, FAV, and FP strains could cause significant elongation of internodes and significant enlargement of the stem nodes, and that all the strains significantly increased the weight of the shoot of *L. chuanxiong*. In addition, FM can significantly increase the LZ coefficient (Table 2). There is no significant difference between the blank group and the treatment group for other growth indicators.

**TABLE 2:** Growth indicators of *L. chuanxiong* for potted verification experiment.

Group	Root	Shoot	Plant height	Crown width	Number of LZ	Diameter of LZ	Diameter of stalk	Coefficient of LZ	Internode length
CK	21.94 ± 5.55	20.38 ± 2.68 a	46.56 ± 6.54	51.06 ± 12.50	55.25 ± 15.86	9.36 ± 1.25 a	4.04 ± 0.40	2.36 ± 0.24 a	4.38 ± 0.77 a
FM	30.75 ± 9.94	33.91 ± 6.74 b	49.50 ± 9.61	54.50 ± 12.33	66.60 ± 16.39	10.38 ± 1.04 b	4.13 ± 0.47	2.58 ± 0.24 b	6.04 ± 0.47 b
FP	29.51 ± 9.13	35.32 ± 6.37 b	48.27 ± 9.67	57.18 ± 15.41	60.00 ± 19.29	10.68 ± 1.28 b	4.47 ± 0.92	2.45 ± 0.30	5.94 ± 0.67 b
FAV	21.13 ± 6.57	37.26 ± 7.25 b	52.10 ± 13.55	54.00 ± 14.90	54.40 ± 9.01	10.43 ± 1.40 b	4.46 ± 0.92	2.40 ± 0.30	6.47 ± 0.97 b

*Means within a column followed by different letters are significantly different (Duncan’s test: P < 0.05).*

#### Determination of Resistant Enzymes

To investigate the effect of the core strains of LZ on the resistance enzyme of *L. chuanxiong*, we measured the resistant enzyme content of the potted *L. chuanxiong*. The production of these resistance enzymes was quantitatively determined by a method described previously (Chen and Wang, 2006). The results showed that compared with the CK group, the strains FM, FAV, and FP all significantly increased the activities of CAT, POD, and PAL in the plants. Compared with FAV, which significantly increased the activity of SOD, FM, and FP significantly reduced the activity of SOD. There is no significant difference between the blank group and the treatment group for MDA (Table 3).

**TABLE 3:** Activities of the protective enzymes for potted verification experiment.

Group	SOD	CAT	MDA	PAL	POD
CK	2.46 ± 0.14 a	2.37 ± 0.17 a	2.50 ± 0.19	3.79 ± 0.02 a	710.50 ± 83.50 a
FM	2.28 ± 0.10 b	6.95 ± 0.34 b	2.58 ± 0.09	4.92 ± 0.04 b	854.17 ± 51.27 b
FP	2.31 ± 0.18 b	8.98 ± 2.02 c	2.26 ± 0.69	4.61 ± 0.02 c	841.67 ± 78.34 b
FAV	2.67 ± 0.17 c	6.27 ± 0.85 b	2.63 ± 0.18	3.98 ± 0.01 d	858.00 ± 80.54 b

*Means within a column followed by different letters are significantly different (Duncan’s test: P < 0.05.*

## Discussion

### Transplanting Reorganizes the Community Structure of Endophytic Fungi

As PX was transplanted from the dam area to the mountain for breeding, the diversity of shared endophytic fungi gradually increased. As PX remained in the dam area for the cultivation of CX, the diversity of shared endophytic fungi gradually decreased. The same rule has been verified in the control breeding experiment. The diversity of endophytic fungi in the plant samples after “breeding on mountains” was significantly higher than that of the plant samples in “dam area breeding.” The endophytic fungal communities of LZ on the mountain of different origins can be distinguished, and the endophytic fungal communities of LZ on the mountain or LZ in the dam area also can be distinguished from the PX of the same period. In contrast, the community structure of endophytic fungi in PX and CX is similar and cannot be distinguished, so do LZ and CX in the dam area. It shows that the transplantation causes the endophytic fungal reorganization of *L. chuanxiong.*

We found that the endophytic fungus of the plant is related to the soil. In the two cultivation modes of the control cultivating experiment, the diversity of endophytic fungi in the LZ samples was significantly higher than that in the CX samples in the same period, so were the corresponding soil samples. The relationship is MLS > MYS > ML > MY in the M-Y mode, and YLS > YYS > YL > YY in the Y-Y mode. In addition, the relative abundance of *Pericona*, *Gibberella*, and *Calophoma* in LZ bred in mountains also showed the same order in the corresponding soil, and the relationship was ML > YL > MLS > YLS. Compared with CX and its soil, LZ and its soil have a higher relative abundance of *Fusarium* (L > Y; LS > YS), while the relative abundance of *Dactylonectria* and *Plectosphaerella* is higher in the CX samples and its soil samples (Y > L, YS > LS). It shows that the recombination of endophytic fungi caused by transplanting is brought by soil microorganisms to a certain extent.

Regardless of “breeding in dam areas (local transplanting)” or “breeding on mountains (remote transplanting),” as long as there is a transplanting process, it can lead to the reorganization of endophytic fungi. However, the endophytic fungal community structure of *L. chuanxiong* not transplanted has no significant changes. It fully shows that change in the breeding environment is the most critical factor for the reorganization of endophytic fungi in *L. chuanxiong.*

### The Recombination of Endophytic Fungi Is Restricted by the Soil Environment, Growth Law of the Host, and the Interaction of Microorganisms

As a result of transplanting, part of the endophytic fungal community structure is reorganized; however, 20–30% of the fungal genera, which belong to the core fungal flora of *L. chuanxiong*, always exist stably whether the plant is transplanting transplanted or not, which belong to the core fungal flora of *L. chuanxiong* and will not disappear because of different growth stages or cultivation areas. Soil is an essential factor in the composition of plant endophytic fungi. Its physical and chemical properties have a decisive effect on the microbial community structure in the rhizosphere and roots of plants (Trivedi et al., 2021). In this study, we found that the main explanatory factors affecting the fungal communities in the rhizosphere and the endophytic environment are not consistent. The main explanatory factor of the rhizosphere flora is available potassium, while that of the endophytic fungi community is hydrolyzed nitrogen. However, there are consistent explanatory factors in the two communities, such as available potassium and organic matter. In addition, we found that some fungi, such as *Phoma*, *Dactylonectria*, and *Rhizoctonia*, showed consistent correlation with environmental factors in the rhizosphere and endophytic states, such as *Phoma*, *Dactylonectria*, and *Rhizoctonia*. It indicates that physiographical factors have different degrees of influence on the rhizosphere and endophytic fungi communities of *L. chuanxiong*, which is consistent with the results of the previous research of Zhang Shuihua (Zhang et al., 2021).

The soil-to-root transfer is one of the most thoroughly studied horizontal transmission routes for endophytes. It shows a great diversity of endophytic communities in the rhizosphere that are still far more diverse than those in the endosphere, and endophytic communities are often a subset of rhizosphere communities (Zhang et al., 2011). In this study, LZ showed a richer diversity of endophytic fungi than CX. On the one hand, the soil environment (including microorganisms and physiographical factors) of *L. chuanxiong* can be changed, and the endophytic fungi will be reshuffled because of the transplantation (Caracciolo and Terenzi, 2021). On the other hand, the relative abundance of some endophytic fungi showed regular changes with plant transplantation and finally existed stably in specific growth stages of *L. chuanxiong* (Zhalnina et al., 2018). At the same time, the endophytic fungal communities tend to be similar, such as in terms of accumulation of pathogenic fungi. For example, *Pericona*, *Gibberella*, etc. (relative abundance increases first, then decreases, and then increases during the transplanting process of FP-FL-SP-SL) are the core flora of LZ, sodopathotroph-saprotroph-symbiotroph. The same result was verified in the control breeding experiment. In this study, it is not difficult to find that the relative abundances of the core flora of LZ, such as *Gibberella*, *Alternaria*, *Periconia*, *Didymella*, and *Calophoma*, are significantly higher in LZ than in CX; the opposite is true in the soil samples. Similarly, the relative abundance of pathotroph-saprotroph in the LZ on the mountain is significantly higher than in the dam area, but there is no significant difference in the corresponding two soils. The relative abundance of *Plectosphaerella* is significantly negatively correlated with *Alternaria* and *Periconia* in the plant samples. However, they are significantly positively correlated in the soil samples. By analyzing the correlation between these fungal communities and physiographical factors, we found that *Gibberella*, *Didymella*, *Calophoma*, *Alternaria*, and *Periconia* exhibited opposite correlations with environmental factors in the rhizosphere and endophytic state, which indicates that the characteristics of some plant endophytic flora tending to be similar with the growth and development of the host are not closely related to the soil, but are regulated by the growth law of the plant host.

In addition, the correlation analysis shows that no matter in plants or soil samples, *Alternaria* and *Periconia* have a significant positive correlation, so do *Gibberella* and *Fusarium*. Some *Fusarium* species have both asexual and sexual reproduction cycles (Leslie and Summerell, 2006). There are at least seven alternative names based on sexual stages related to *Fusarium*, and *Gibberella* is one of them (Geiser et al., 2013). It implies an interaction between endophytic fungi, which can attract the other party to colonize the *L. chuanxiong* tissue, but further research is needed.

### Remote Transplantation Is an Important Opportunity to Reshuffle the Micro-Ecological Structure of Asexually Propagated Plants

There are abundant pathogenic fungi in all the stages of *L. chuanxiong* and its soil. On the one hand, transplantation shortens the interaction time between the host and various pathogens and reduces the incidence of *L. chuanxiong*. On the other hand, transplanting will cause the emergence of susceptible wounds and change the soil environment, which increases the chances of plants being infected with other pathogens. The situation often occurs in the form of grape black foot disease (Whitelaw-Weckert et al., 2013). We find that in the plant samples, pathotroph is more abundant in the samples from the dam areas and less in the samples from the mountains, which shows that “breeding on mountains” can reduce the colonization of plant pathogens.

The interaction between plants and the associated microbiome may be deleterious, beneficial, or neutral to the host. Phytopathogenic fungi usually refer to microorganisms that could cause the host to suffer from diseases, such as wilt and root rot (Ma et al., 2013; Latz et al., 2018). Due to host specificity, although pathogenic fungi are harmful to the host, they may be beneficial or neutral microbial communities for other plants, and some pathogenic fungi have different host specificities for the same strain due to different gene regions (Ma et al., 2013; Zhang and Ma, 2017; de Lamo and Takken, 2020). Beneficial or neutral endophytes can directly interact with pathogens through parasitism, antibacterial effects, or competition for nutrients or root niches. They can also indirectly confer biological control by inducing host resistance mechanisms and even synthesize some chemical substances to regulate the growth of the host (Wiewiora et al., 2015; Constantin et al., 2020). Most of the “potential pathogens” in *L. chuanxiong* may belong to non-transformed phytopathogenic fungi. Their effects on *L. chuanxiong* are not deleterious. Instead, they regulate or balance the symbiotic relationship between plants and pathogens.

The pot experiment has proved the point. In a large body of literature, the three strains used for pot experiment are identified as pathogenic fungi, whether they were *Fusarium* in the sexual reproduction stage (Ma et al., 2013) or *Gibberella* in the asexual reproduction stage (Geiser et al., 2013), which can cause a variety of plant diseases. All the strains could produce gibberellin and auxin at the same time, and increase the shoot, diameter of the stem node, and length of internodes of *L. chuanxiong*. The diameter of the stem node is an important reference factor to evaluate the quality of LZ. The larger the diameter of the stem nodes is, the better the quality of LZ (seed). The coefficient of LZ$(\mathrm{Coefficient}\mathrm{of}\mathrm{LZ}=\frac{\mathrm{The}\,\mathrm{diameter}\,\mathrm{of}\,\mathrm{node}}{\mathrm{The}\,\mathrm{diameter}\,\mathrm{of}\,\mathrm{stalk}}$) is also an important factor in evaluating the quality of LZ. In this study, FM can cause a significant increase in the coefficient of LZ. In addition, we found that FM, FAV, and FP can increase the activity of *L. chuanxiong*-resistant enzymes, such as CAT, POD, and PAL, indicating that they can all improve the defensive ability of *L. chuanxiong*. In the research results, we also found that the LZ cultivated in the mountainous area separated more gibberellin and auxin-producing strains than the LZ cultivated in the dam area, which indicates that there are indeed more growth-promoting fungi in the mountain environment.

We find that the relative abundance of some dominant genera in LZ will increase significantly as long as transplanting occurs. However, the relative abundance of these genera in transplanting on the mountain is significantly higher than in transplanting in the dam area and non-transplanting. Some of the dominant genera in CX only increase when transplanted in the mountains. Some genera increased when transplanted in mountainous areas, and the relative abundance does not change significantly when transplanted in the dam area or without transplanting.

Regardless of breeding in mountains or breeding in the flat dam, recombination will occur as long as there is a transplanting process. The transplantation changes the chance of pathogen colonization in plant hosts, reduces the time for pathogens to interact with the host, and increases the colonization of beneficial or neutral endophytes. Plant pathogenic, beneficial, and neutral fungi have all obtained the opportunity to reshuffle during the recombination process. Remote transplantation brings more significant reorganization than local transplantation, and this reorganization is mainly reflected in the reduction of pathogenic fungi and the acquisition of core flora. It fully shows that remote transplantation is an important opportunity for reshuffling the micro-ecological structure of asexually propagated plants, which is an important process for the asexual reproduction of species to “rejuvenate.”

### Rhizosphere Microbiome Assembly Results From the Combination of Microbial Substrate-Utilization Traits and Plant-Root Exudation

The plant microbiome comprises the rhizosphere, phyllosphere, and endosphere, which is a microhabitat, comprising roots and the 1–2 mm soil immediately surrounding them (Sasse et al., 2018). The rhizosphere microbiome plays an important role in nutrient cycling, soil fertility maintenance, and carbon sequestration, directly and indirectly affecting the health of animals and plants in different ecosystems (Jia et al., 2018). Ecosystems based in the soil microbiome are influenced and controlled by multiple abiotic and environmental factors, such as soil structure and type, soil pH, soil nutrients, geographical factors (altitude, latitude, and longitude), climatic factors (UV radiation, CO_2_, and temperature) (Islam et al., 2020). Abiotic and environmental factors result in the establishment of multiple microenvironments that offer a diversity of ecological niches (Caracciolo and Terenzi, 2021). In the previous experiments, we found that the diversity of species, main ecological functional groups, and differences in production areas of the fungal community showed a gradual change pattern between the non-rhizosphere soil, the rhizosphere soil, and the rhizome of *L. chuanxiong*, which proved that during the cultivation process, plants obtain part of the species from the soil of the production area and accumulate or subtract continuously to form a specific endophytic flora (Zhang et al., 2021). On the one hand, it showed that there was a horizontal relationship between the three communities. On the other hand, it also showed that the higher the degree of microenvironmental specialization, the stronger the selectivity and enrichment of microorganisms (Zhang et al., 2021). In this research, the explanatory factors affecting the community structure of fungi in the rhizosphere include available potassium, available phosphorus, organic matter, and hydrolyzed nitrogen, and available potassium is the most important explanatory factor.

There is an intense chemical interaction between plants and microorganisms. In the rhizosphere, plants release root exudates, which promote microorganism population development. The rhizosphere offers varieties of carbon-rich micro-habitats, and beneficial microorganism populations can colonize by using such substrates (Caracciolo and Terenzi, 2021). Plants contribute to the assemblage of their rhizobiome (Mercado-Blanco et al., 2018; Sasse et al., 2018; Poveda et al., 2020), and some plant species can have their specific microbial community (Mhlongo et al., 2018), which can, in turn, change during their growing stage, and in different root regions, the results of the LZ(CX) rhizosphere fungal communities tending to be similar in this study are consistent with this view. The composition of root exudates may be different and lead to differences in rhizosphere microbial populations, depending on plant species, growth phases, exposure of a plant to stress conditions, and, sometimes, differences in plants of the same species (Sasse et al., 2018; Zhalnina et al., 2018; Caracciolo and Terenzi, 2021). For example, studies have found that rhizosphere communities in heavy metal (HM)-contaminated soil are crop-specific (Sun et al., 2018). In addition, phytohormones are involved in plant growth and stress response, as well as rhizosphere communication (Leach et al., 2017). The production and release of phytohormones are crucial to the combination of root biomes, for they can be used by microorganisms as signal molecules and carbon and nitrogen sources (Shahzad et al., 2016). Plant- microbial symbiosis cannot only produce exogenous plant hormones but also regulates the production of endogenous plant hormones, indicating that there is crosstalk between plants and microorganisms (Shahzad et al., 2016). Phytohormones (such as salicylic acid, jasmonic acid, and ethylene) and resistance enzymes act mostly in an antagonistic manner, leading to tradeoffs for resistance against organisms of diverse lifestyles, and are the main regulators of plant response to an attack by pathogens and pests (Leach et al., 2017).

Plant-associated microorganisms use chemotaxis to sense and respond to plant-derived signals, such as organic acids and sugars present in plant exudates. Once a signal is perceived, microorganisms move toward the plant to initiate colonization. Polyamines function as signaling molecules in the root–rhizosphere interface and inform the microbiome of the presence of eukaryotic hosts. Sensing of such molecules triggers a lifestyle switch to promote attachment and biofilm formation in many microbial groups (Jimenez-Bremont et al., 2014; Liu et al., 2018). After successful colonization, diverse host processes can lead to different niche colonization patterns among different microbial groups, such as the activation of plant signaling pathways and/or nutrient stress-mediated root inhibition that alters the architecture of the host root (Trivedi et al., 2021). The presence of antibiotic resistance genes may provide protection against biological and chemical warfare that occurs during the initial stages of community assembly.

Arbuscular mycorrhizal fungi and rhizobia can also affect the rhizosphere microbial community. Mycorrhizal plants displayed a preferential association with Comamonadaceae, Oxalobacteraceae, and *Rubrivivax* spp., as well as an enrichment of the type III secretion system (T3SS)-carrying *Pseudomonas* spp., relative to non-mycorrhizal plants (Viollet et al., 2011; Uroz et al., 2019). Mycorrhizal fungi exert an influence on the surrounding plant environment through the direct action of the fungal mantle formed around the roots, which changes soil properties and metabolites around the roots, and, consequently, the recruitment of microorganisms to the hyphal network. Some studies also reported that AM fungal communities differed between roots with and without nodules (Uroz et al., 2019).

### Recombination of Endophytic Fungi Regulates Plant Growth, Development, and Resistance

Plants provide a multitude of niches for the growth and proliferation of a diversity of microorganisms. These microorganisms can form complex co-associations with plants and have important roles in promoting the productivity and health of plants in natural environments (Coats and Rumpho, 2014; Salazar-Cerezo et al., 2018). Some microorganisms can produce secondary metabolites with antimicrobial activity or synthesize different plant hormones, such as gibberellins (Tsavkelova et al., 2008; Tsavkelova, 2016), to participate in plant growth and development. In this study, there are indeed more gibberellin- and auxin-producing fungi in the mountain environment than in the dam area. It has been found that *Gibberella* can synthesize gibberellins and participate in plant growth and development, such as seed germination, stem elongation, flowering, and fruit development, etc. (Ma et al., 2013; Hedden and Sponsel, 2015). *Ilyonectria* and *Dactylonectria* are pathogenic fungi of plants, which can lead to the decline of young plants. Characteristic symptoms include low fruit yields, very short shoots, and severely stunted roots with few feeder roots and black sunken necrotic lesions (Whitelaw-Weckert et al., 2013; Agusti-Brisach et al., 2016; Manici et al., 2018; Wakelin et al., 2018; Juroszek et al., 2020). It revealed more *Gibberella* in LZ, and *Ilyonectria* and *Dactylonectria* gradually increase as CX matures. In breeding on mountains, *Gibberella* advances the gibberellin available for *L. chuanxiong* and regulates plant growth. The gibberellin available for *L. chuanxiong* regulates plant growth. At the same time, the decrease of *Ilyonectria* and *Dactylonectria* leads to the reduction of factors that inhibit the degradation of *L. chuanxiong*, but this requires further research. Our results have confirmed our conjecture about *Gibberella*, but since *Ilyonectria* and *Dactylonectria* have not been isolated, we cannot confirm the effects of these two genera on the growth of *L. chuanxiong* plants. We will further study in the later stage.

Plant-related microorganisms help maintain plant biodiversity through different biological processes (Hardoim et al., 2015) and promote pathogen resistance (Hajiboland et al., 2010; Huang et al., 2010; Khalloufi et al., 2017) and abiotic stress resistance (de Lamo et al., 2018; de Lamo and Takken, 2020). Research suggests that endophytes of the same genus have similar ecological niches and antagonize each other in the same environment (Torres et al., 2012). Endophytes that preemptively occupy ecological sites in the internal environment of a plant can hinder and prevent the invasion and colonization of similar pathogens (Torres et al., 2012). It has been found that the LZ in the mountain cultivation stage carries a variety of endophytes (Li, 2016) that can antagonize the root rot of *L. chuanxiong* (Lin, 2017). Fungi infected during breeding on mountains can spread vertically to CX through LZ. For CX cultivated in the dam area, these strains can act as preconceived niche occupants. They may also be a nutritional competitor of susceptible pathogens in the flat dam stage to ensure the disease resistance of *L. chuanxiong* during the cultivation period in the flat dam (Yuliar et al., 2015; Schlatter et al., 2017). Endophytic fungi are different from pathogenic fungi, such as Streptomyces in LZ (He et al., 2016; Lin et al., 2017). These endophytic strains directly inhibit or kill the synthesis of pathogenic fungi by releasing antibiotics, bactericidal proteins, chitinase, and other chemical substances. It has been found that the endophytic fungi of *L. chuanxiong* are often significantly related to their active ingredients. For example, the content of ibutilide is significantly related to endophytes, such as *Penicillium* and *Wardell*, and *Bacillus* subtilis can secrete Ligustrazine (Yin et al., 2018a,b, 2019). Therefore, endophytic fungi can affect the quality of the rhizome of *L. chuanxiong* by regulating the production of its secondary metabolites.

## Conclusion

Our results show that transplantation leads to the reorganization of the endophytic fungus structure of *L. chuanxiong*, and that remote transplantation brings more significant reorganization than local transplantation, indicating that the mountain environment contributes more to the reorganization process. The phenomenon is mediated by the soil environment of *L. chuanxiong* and the growth pattern of plants. In addition, we believe that breeding on mountains provides an opportunity for the reorganization of endophytic fungi to *L. chuanxiong*. The soil environment has increased the types of endophytic fungi in *L. chuanxiong*, and the community structure has become more complicated. Repeated transplanting also reduced the opportunity and time of interaction between *L. chuanxiong* and pathogenic fungi. It increased the probability of colonization of beneficial fungi and neutral fungi. Therefore, it can regulate the disease resistance of plants and then affect the quality of LZs. Although our research is limited to a single species and more in-depth research is needed to clearly understand the mechanism of asexual reproduction of plants, our results also have important practical significance for studying the mechanism of “breeding on mountains” to avoid the complications of asexual reproduction of *L. chuanxiong*. It also provides ideas for solving the mechanism research of other asexual reproduction plants. The research on the micro-ecological mechanism of medicinal plants or economic plants also has critical enlightenment. In addition, our research also provides a natural example for the construction of an apomictic species.

## Data Availability Statement

The datasets presented in this study can be found in online repositories. The names of the repository/repositories and accession number(s) can be found below: NCBI BioProject. accession PRJNA778178.

## Author Contributions

ZY: conceptualization. DH: methodology. GH, HL, and WX: resources. LK: writing—original draft preparation and writing—review and editing. DH, ZY, and LH: supervision, project administration, and funding acquisition. ZZ: participated in the experimental work. All authors have read and agreed to the published version of the manuscript.

## Conflict of Interest

The authors declare that the research was conducted in the absence of any commercial or financial relationships that could be construed as a potential conflict of interest.

## Publisher’s Note

All claims expressed in this article are solely those of the authors and do not necessarily represent those of their affiliated organizations, or those of the publisher, the editors and the reviewers. Any product that may be evaluated in this article, or claim that may be made by its manufacturer, is not guaranteed or endorsed by the publisher.

## Footnotes

1
https://www.mothur.org/wiki/Download_mothur
