# Supplementary material for: “Breeding on Mountains” Resulted in the Reorganization of Endophytic Fungi in Asexually Propagated Plants (Ligusticum chuanxiong Hort.)
Source: Front Plant Sci. 2021 Nov 10;12:740456. doi: 10.3389/fpls.2021.740456 (PMC8631752; doi:10.3389/fpls.2021.740456)
Supplement: Supplementary file 1 [file Data_Sheet_1.docx]

Supplementary Material

**Supplementary** **Figure**


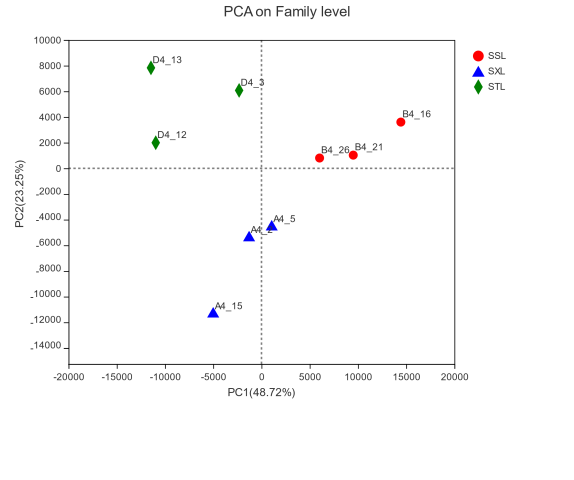

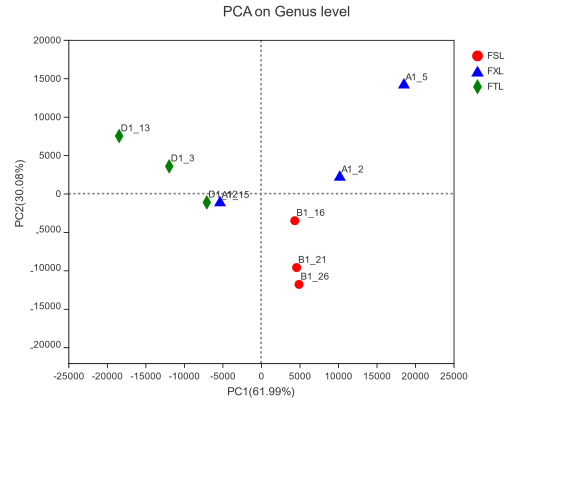

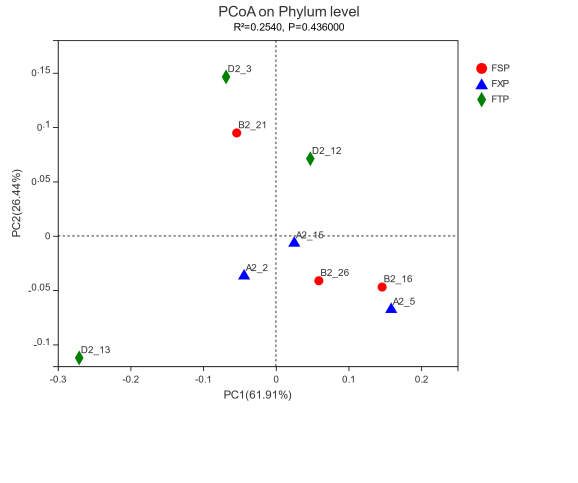

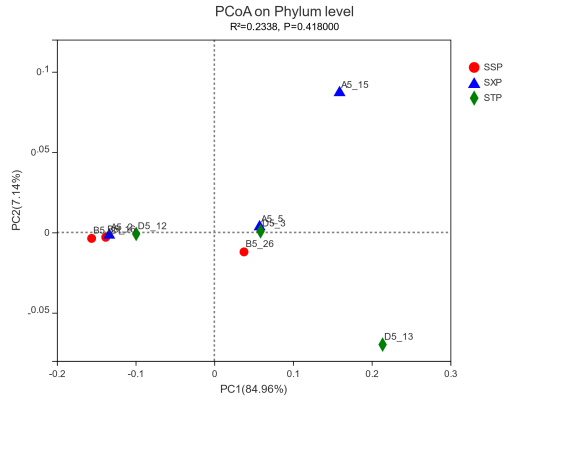

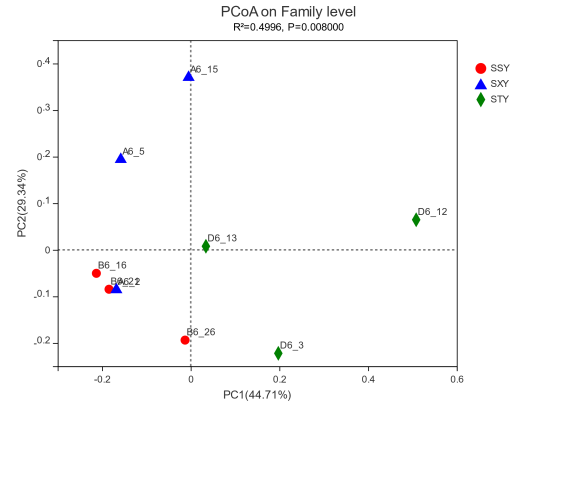

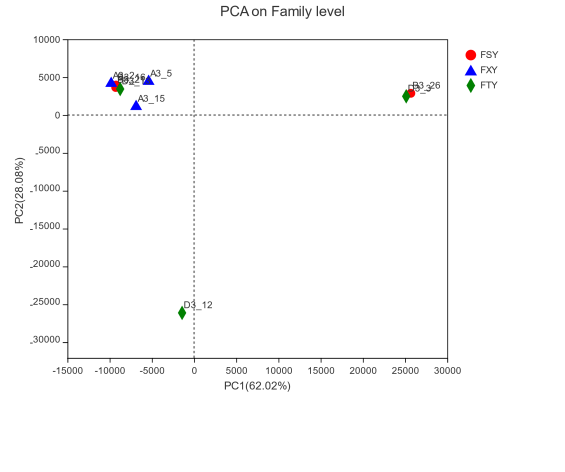


A

B

C

D

E

F

Figure S1 PCA image of endophytic fungi of Lingzi in the first year A: LZ in the first cultivation cycle;B: LZ in the second cultivation cycle;C: PX in the cultivation cycle; D: PX in thecultivation cycle;E: CX in the cultivation cycle;F: CX in the second cultivation cycle.


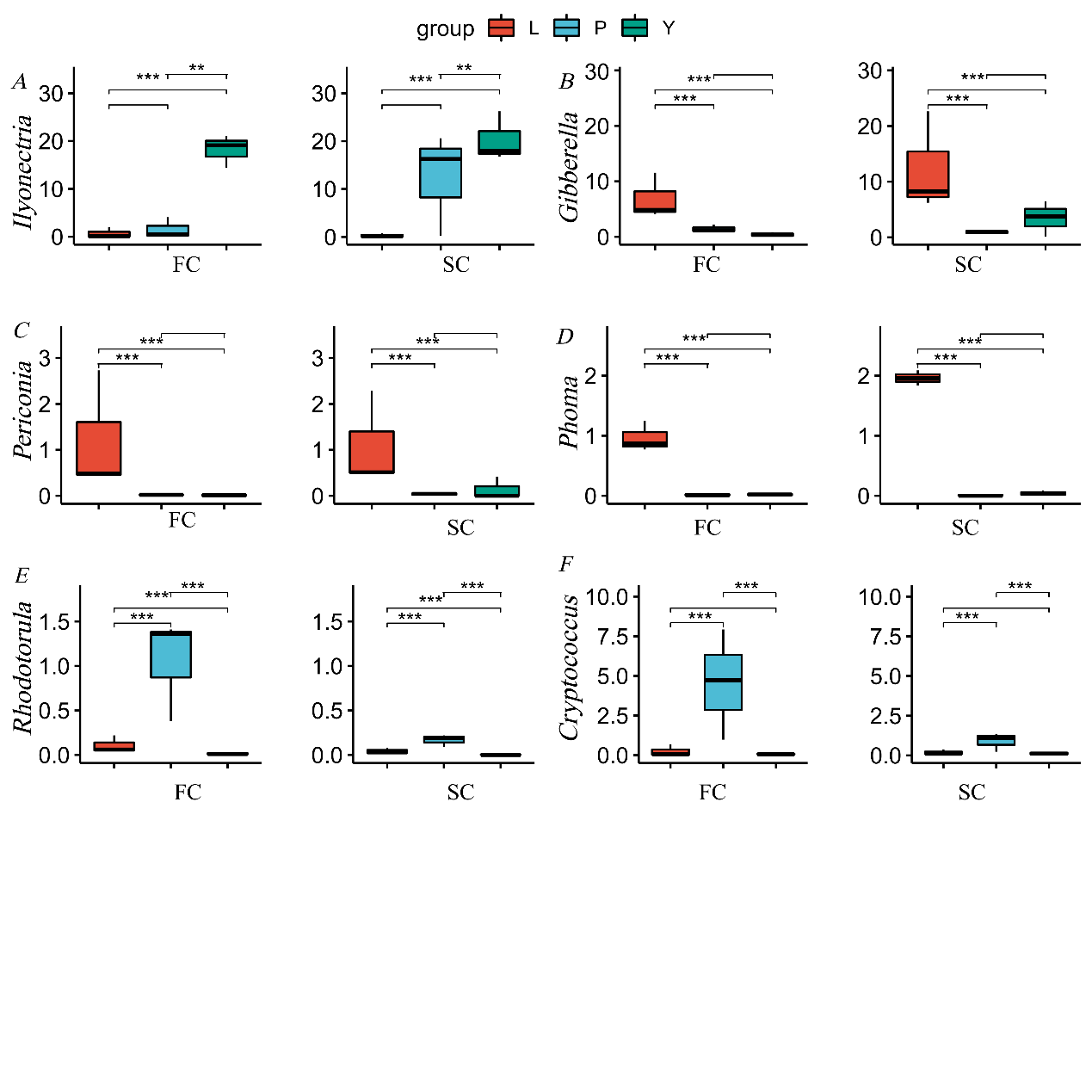


Figure S2 Significantly represented fungal taxa.FC refer to the first cultivation cycle;SC refer to the second cultivation cycle A *Ilyonectria*; B *Gibberella;*C *Pericona;*D *Phoma;*E *Rhodotorula;*F *Cryptococcus.*. Whiskers denote standard errors of the mean. Significant differences (P < 0.05), assessed using robust ANOVA and Tukey’s HSD test, are indicated by asterisks.


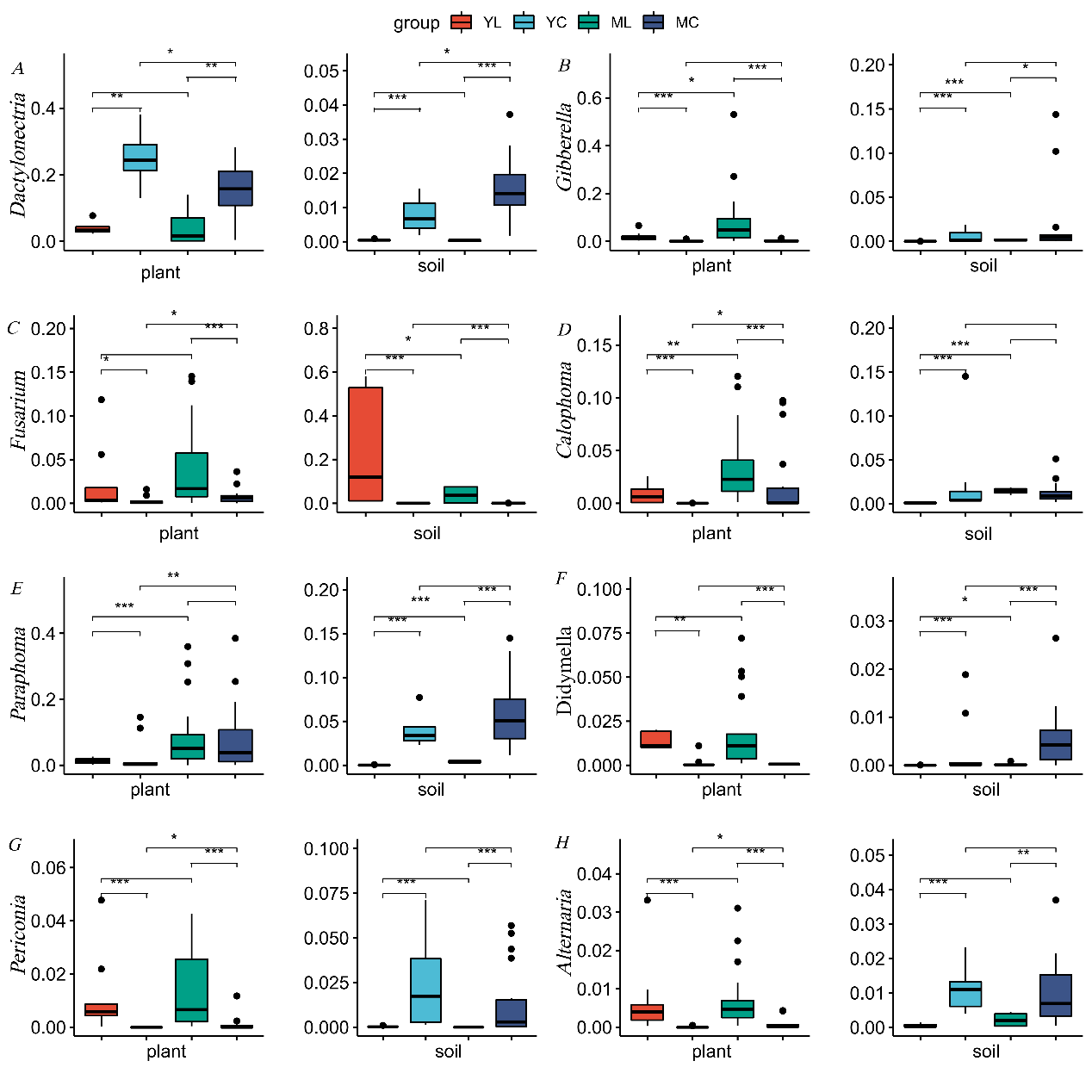


Figure S3 Significantly represented fungal taxa. A Dactylonectria; B Gibberella;C Fusarium；D Calophoma;E Paraphoma;F Didymella;G Pericona;H Alternaria. arenariae. Whiskers denote standard errors of the mean. Significant differences (P < 0.05), assessed using robust ANOVA and Tukey’s HSD test, are indicated by asterisks.


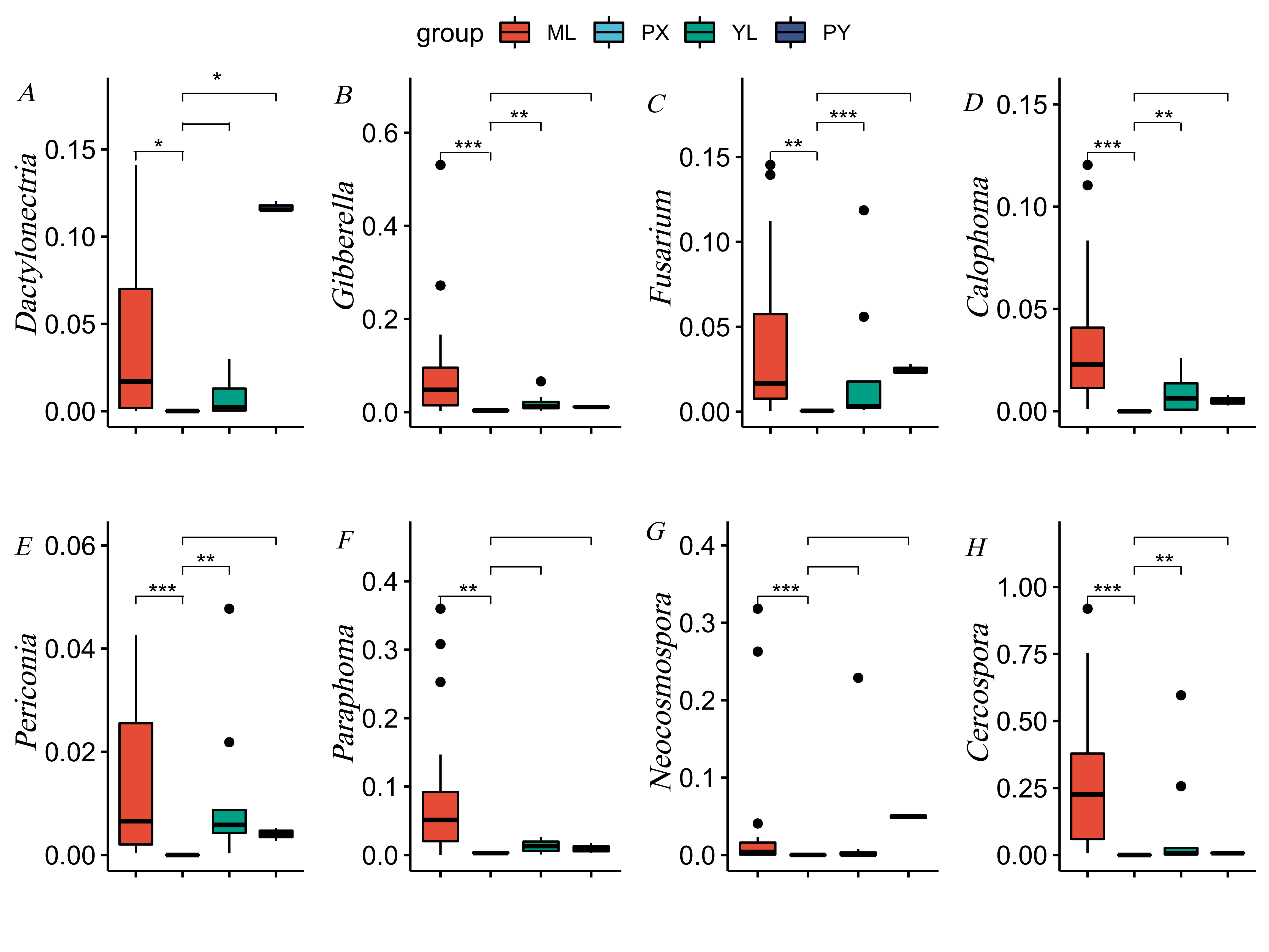


Figure S4 Significantly represented fungal taxa. ML refer to Lingzi under M-Y cultivation modes;YL refer to Lingzi under Y-Y cultivation modes；PX refer to the Provenance of Lingzi Puxiong;PY refer to CX without transplantation.


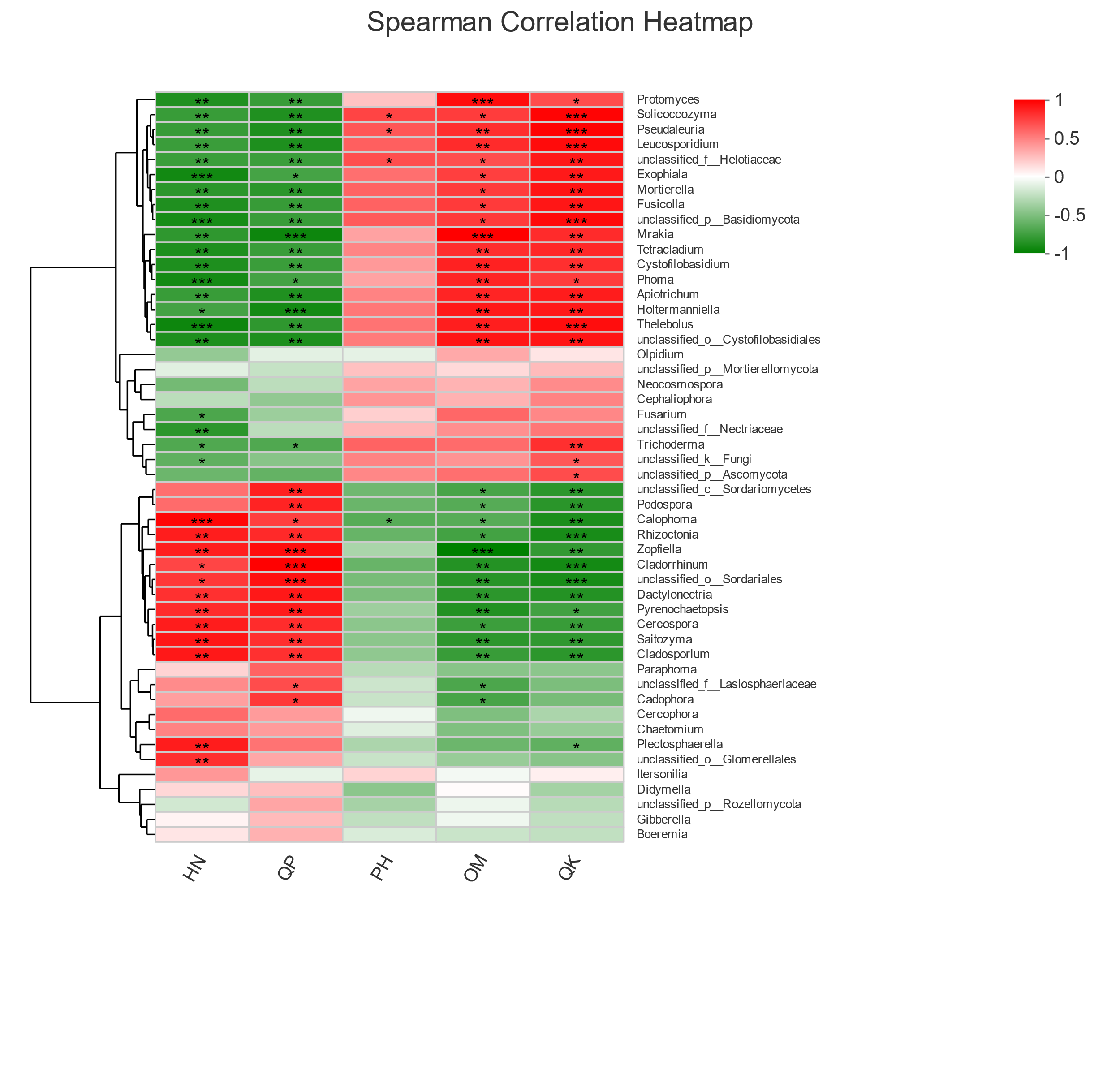

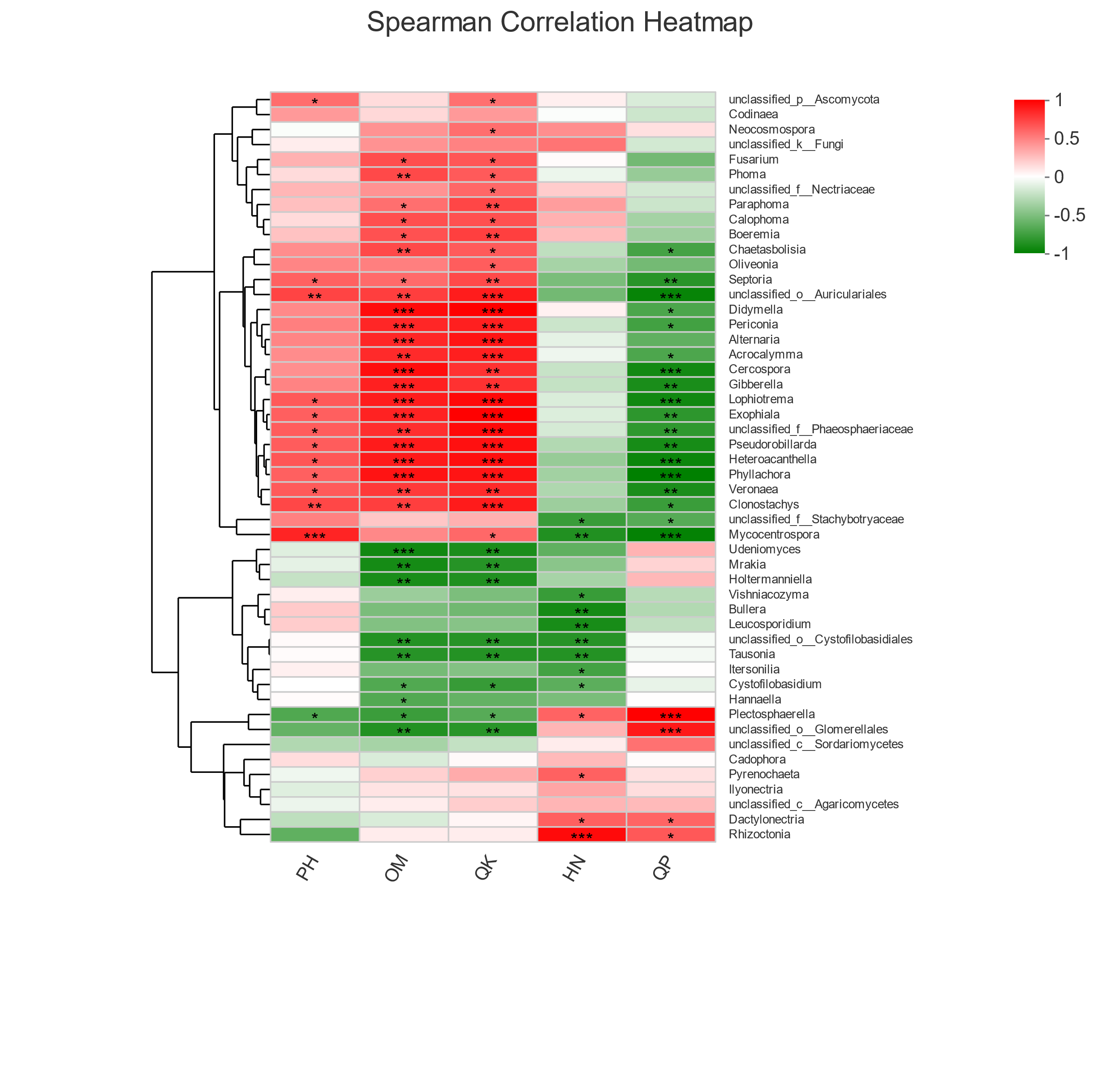
Figure S5 A. Spearman correlation heatmap between endophytic fungi and soil physical and chemical factors in the rhizosphere. B. Spearman correlation heatmap between rhizosphere fungi and rhizosphere soil physical and chemical factors.

B

A

**Supplementary** **Table**

Table S1 Number of experimental materials for cycling cultivation experiment

| **Time** | **Organs** | **Stages of cultivation** | **Breeding base** | **Number** | | |
| --- | --- | --- | --- | --- | --- | --- |
|  |  |  |  | **Place** | **Year/place** | **Year** |
| The first round of cultivation (F) | stem node | Lingzi  (LZ, L) | Xiaoyudong | X1-1, X1-2, X1-3 | FXL | FL |
|  |  |  | Shuimo | S1-1, S1-2, S1-3 | FSL |  |
|  |  |  | Taian | T1-1, T1-2, T1-3 | FTL |  |
|  | rhizome | Puxiong  (PX, P） | Xiaoyudong | X2-1, X2-2, X2-3 | FXP | FP |
|  |  |  | Shuimo | S2-1, S2-2, S2-3 | FSP |  |
|  |  |  | Taian | T2-1, T2-2, T-2-3 | FTP |  |
|  |  | Chuanxiong  (CX, Y) | Xiaoyudong | X3-1, X3-2, X3-3 | FXY | FY |
|  |  |  | Shuimo | S3-1, S3-2, S3-3 | FSY |  |
|  |  |  | Taian | T3-1, T3-2, T3-3 | FTY |  |
| The second round of cultivation (S) | stem node | Lingzi  (LZ, L) | Xiaoyudong | X4-1, X4-2, X4-3 | SXL | SL |
|  |  |  | Shuimo | S4-1, S4-2, S4-3 | SSL |  |
|  |  |  | Taian | T4-1, T4-2, T4-3 | STL |  |
|  | rhizome | Puxiong  (PX, P） | Xiaoyudong | X5-1, X5-2, X5-3 | SXP | SP |
|  |  |  | Shuimo | S5-1, S5-2, S5-3 | SSP |  |
|  |  |  | Taian | T5-1, T5-2, T5-3 | STP |  |
|  |  | Chuanxiong  (CX, Y) | Xiaoyudong | X6-1, X6-2, X6-3 | SXY | SY |
|  |  |  | Shuimo | S6-1, S6-2, S6-3 | FSY |  |
|  |  |  | Taian | T6-1, T6-2, T6-3 | FTY |  |
|  |  |  | Meishan | MW | Breeding base is unknown | |
|  |  |  | Aoping | PA |  |  |
|  |  |  | Shiyang | DS |  |  |
|  |  |  | Xiaoyudong | PX |  |  |

Table S2 Number of experimental materials for comparative breeding experiment between mountainous and flat dam

| **Cultivation mode** | **Stages of cultivation** | **Time** | **Origin** | **Number** | |
| --- | --- | --- | --- | --- | --- |
|  |  |  |  | **Plant** | **Soil** |
| M-Y | Puxiong | The second round of cultivation | Shiyang | PX | YPG |
| M-Y | Chuanxiong | The second round of cultivation | Shiyang | PY |  |
| M-Y | Lingzi | The third round of cultivation | Shuimo | ML | MLS |
| M-Y | Chuanxiong | The third round of cultivation | Shiyang | MY | MYS |
| Y-Y | Lingzi | The third round of cultivation | Shiyang | YL | YLS |
| Y-Y | Chuanxiong | The third round of cultivation | Shiyang | YY | YYS |

Table S3 Number of experimental materials for potted verification experiment

| **Group** | **Function** | | **Approach** | **Cycle** |
| --- | --- | --- | --- | --- |
|  | **GA** | **IAA** |  |  |
| FM | + | + | 60ml 5×10^8^ spore suspension is used to irrigate roots, | Two weeks |
| FP | + | + | 60ml 5×10^8^ spore suspension is used to irrigate roots | Two weeks |
| FAV | + | + | 60ml 5×10^8^ spore suspension is used to irrigate roots | Two weeks |
| CK | - | - | 60ml sterile PDB culture medium is used to irrigate the roots | Two weeks |

Table S4 Richness and Diversity in endophytic fungi

of *Ligusticum chuanxiong* in different periods

| **Sample** | **OTU** | | **ace** | **coverage** | | **shannon** |
| --- | --- | --- | --- | --- | --- | --- |
| LZ | 131 | 156 | | 0.999312 | 2.19 | |
| PX | 110 | 143 | | 0.999303 | 1.61 | |
| CX | 91 | 116 | | 0.999355 | 1.41 | |

Table S5 Richness and Diversity of Endophytic Fungi in Plants and Soil Samples under Different Cultivation Modes

| **Sample** | **OTUs** | **ace** | **coverage** | **shannon** |
| --- | --- | --- | --- | --- |
| ML | 318 | 129 | 0.999364 | 2.19 |
| MY | 256 | 63 | 0.999778 | 1.46 |
| YL | 258 | 108 | 0.999565 | 1.63 |
| YY | 141 | 63 | 0.999727 | 1.11 |
| PX | 98 | 84 | 0.999552 | 2.05 |
| PY | 235 | 198 | 0.998998 | 1.77 |
| YLS | 538 | 250 | 0.998875 | 2.70 |
| YYS | 575 | 245 | 0.998725 | 2.85 |
| MLS | 848 | 318 | 0.998661 | 3.43 |
| MYS | 629 | 278 | 0.998592 | 3.02 |

Table S6 RDP analysis table of soil physical and chemical factors

|  | **Rhizosphere** | | **Endophytes** | |
| --- | --- | --- | --- | --- |
|  | **r2** | **p_values** | **r2** | **p_values** |
| PH | 0.3856 | 0.107 | 0.3240 | 0.104 |
| OM | 0.9650 | 0.001 | 0.9458 | 0.001 |
| HN | 0.8222 | 0.001 | 0.9909 | 0.001 |
| QP | 0.9649 | 0.002 | 0.9105 | 0.001 |
| QK | 0.9681 | 0.002 | 0.9520 | 0.001 |

Table S7 Capability of some fungal isolates to produce gibberellins and auxins

| **LZ in the mountains** | | | **LZ in the dam areas** | | |
| --- | --- | --- | --- | --- | --- |
| **Number** | **GA** | **IAA** | **Number** | **GA** | **IAA** |
| M-272 | - | + | S-69 | - | + |
| M-192 | - | + | S-107 | - | + |
| M-227 | - | + | S-106 | - | + |
| M-121 | + | + | S-90 | - | + |
| M-252 | + | + | S-59 | - | + |
| M-313 | + | + | S-23 | - | + |
| M-320 | - | + | S-19 | - | + |
| M-228 | - | + | S-52 | - | + |
| M-203 | - | + | S-60 | - | + |
| M-237 | - | + | S-25 | + | - |
| M-279 | - | + | S-18 | + | - |
| M-311-1 | - | + | S-4 | + | - |
| M-341-1 | + | + | S-17 | + | - |
| M-341-2 | + | - | S-69 | + | - |
| M-340-2 | + | - | S-113 | + | - |
| M-311-2 | + | - | S-12 | + | - |
| M-198 | + | - |  |  |  |
| M-207 | + | - |  |  |  |
| M-201 | + | - |  |  |  |
| M-319 | + | - |  |  |  |
| M-301 | + | - |  |  |  |
| M-249 | + | - |  |  |  |
| M-379 | + | - |  |  |  |
| M-232 | + | - |  |  |  |
